# Probing the oxidation state of transition metal complexes: a case study on how charge and spin densities determine Mn L-edge X-ray absorption energies†
†Electronic supplementary information (ESI) available. See DOI: 10.1039/c8sc00550h


**DOI:** 10.1039/c8sc00550h

**Published:** 2018-07-17

**Authors:** Markus Kubin, Meiyuan Guo, Thomas Kroll, Heike Löchel, Erik Källman, Michael L. Baker, Rolf Mitzner, Sheraz Gul, Jan Kern, Alexander Föhlisch, Alexei Erko, Uwe Bergmann, Vittal Yachandra, Junko Yano, Marcus Lundberg, Philippe Wernet

**Affiliations:** a Institute for Methods and Instrumentation for Synchrotron Radiation Research , Helmholtz-Zentrum Berlin für Materialien und Energie GmbH , Albert-Einstein-Strasse 15 , 12489 Berlin , Germany . Email: wernet@helmholtz-berlin.de; b Department of Chemistry-Ångström Laboratory , Uppsala University , Sweden . Email: marcus.lundberg@kemi.uu.se; c SSRL , SLAC National Accelerator Laboratory , Menlo Park , California 94025 , USA; d Institute for Nanometre Optics and Technology , Helmholtz-Zentrum Berlin für Materialien und Energie GmbH , Albert-Einstein-Strasse 15 , 12489 Berlin , Germany; e The School of Chemistry , The University of Manchester at Harwell , Didcot , OX11 OFA , UK; f Molecular Biophysics and Integrated Bioimaging Division , Lawrence Berkeley National Laboratory , Berkeley , California 94720 , USA; g LCLS , SLAC National Accelerator Laboratory , Menlo Park , California 94025 , USA; h Institut für Physik und Astronomie , Universität Potsdam , Karl-Liebknecht-Strasse 24/25 , 14476 Potsdam , Germany; i Stanford PULSE Institute , SLAC National Accelerator Laboratory , Menlo Park , California 94025 , USA

## Abstract

Transition metals in inorganic systems and metalloproteins can occur in different oxidation states, which makes them ideal redox-active catalysts. To gain a mechanistic understanding of the catalytic reactions, knowledge of the oxidation state of the active metals, ideally *in operando*, is therefore critical. L-edge X-ray absorption spectroscopy (XAS) is a powerful technique that is frequently used to infer the oxidation state *via* a distinct blue shift of L-edge absorption energies with increasing oxidation state. A unified description accounting for quantum-chemical notions whereupon oxidation does not occur locally on the metal but on the whole molecule and the basic understanding that L-edge XAS probes the electronic structure locally at the metal has been missing to date. Here we quantify how charge and spin densities change at the metal and throughout the molecule for both redox and core-excitation processes. We explain the origin of the L-edge XAS shift between the high-spin complexes Mn^II^(acac)_2_ and Mn^III^(acac)_3_ as representative model systems and use *ab initio* theory to uncouple effects of oxidation-state changes from geometric effects. The shift reflects an increased electron affinity of Mn^III^ in the core-excited states compared to the ground state due to a contraction of the Mn 3d shell upon core-excitation with accompanied changes in the classical Coulomb interactions. This new picture quantifies how the metal-centered core hole probes changes in formal oxidation state and encloses and substantiates earlier explanations. The approach is broadly applicable to mechanistic studies of redox-catalytic reactions in molecular systems where charge and spin localization/delocalization determine reaction pathways.

## Abstract 2

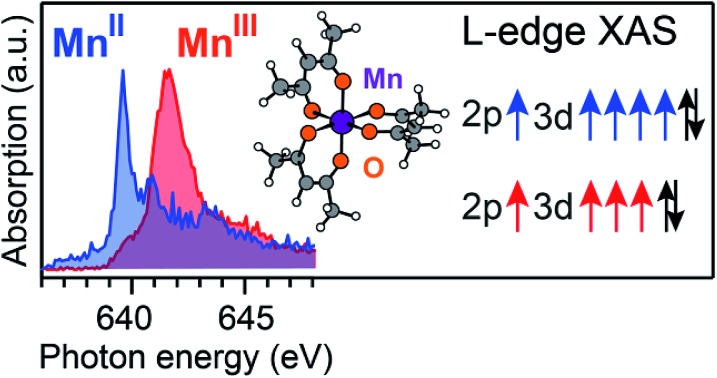
A combined experimental and theoretical approach reveals correlations of metal L-edge X-ray absorption energies to local charge and spin densities.

## Introduction

The mechanistic understanding of catalytic reactions involving 3d transition metals is an essential goal in a wide range of research in materials science, inorganic chemistry and biochemistry, including photocatalysis, electrocatalysis and enzymology.^1^–^10^ Reaction mechanisms are often described in terms of changes of oxidation and spin states of the 3d metal, and to discriminate between alternative mechanisms, experimental and theoretical methods are required that can quantitatively characterize these properties.

Many different experimental techniques are used to determine the spin and oxidation states of the metal centers in molecular complexes. Spin states can be determined using magnetic resonance methods such as electron paramagnetic resonance (EPR), and knowing if a system is in a high-spin or low-spin configuration can then be used to assign its oxidation state. Another powerful method to probe the oxidation state, which is directly connected to the charge density of metals, is using X-ray spectroscopy, including X-ray absorption spectroscopy (XAS) also termed X-ray absorption near edge spectroscopy (XANES) and X-ray emission spectroscopy (XES). Among X-ray spectroscopy, XAS and XES studies at the 3d-metal K-edges with hard X-rays are more commonly used^1^,^11^–^17^ for chemical and biochemical systems, while L-edge XAS and XES in the soft X-ray range are more often used for materials science. L-edge XAS has the advantage to directly probe the metal-derived 3d valence orbitals *via* the dipole-allowed 2p–3d transitions.^13^,^18^–^23^ Compared to K-edge spectroscopy it also has a higher spectral sensitivity (less core–hole lifetime broadening), but is technically more challenging, because of the more restricted sample environment and strong X-ray induced sample damage for sensitive molecular complexes and biological samples. L-edge XAS of 3d-transition metal systems typically shows distinct changes in spectral shape and incident energy with changes in the metal oxidation state. For high-spin metal complexes the L-edge spectrum shifts to higher energy with increasing formal metal oxidation state and shows significant changes in spectral shape.^13^,^22^,^24^–^28^

An alternative way to describe oxidation states of metal centers in molecular complexes is to calculate the charge and spin density distributions in the system. Quantum chemistry calculations indicate that the local charge density on the 3d transition metal atom or ion in a complex does not strongly correlate with its formal oxidation state while spin density is localized at the metal.^29^,^30^ One systematic study of a series of Mn complexes has suggested that oxidation-state changes do not occur on the Mn atoms but on the ligands.^31^ In another study the authors have concluded that “reduction or oxidation of the molecule is therefore not a reduction or oxidation locally of the metal ion but of the whole molecule”.^29^ This picture of almost complete delocalization of valence charge needs to be reconciled and unified with the often used notion that L-edge XAS is a local probe of the charge density at the absorbing metal. Thus, we need a better description of how charge and spin densities are reflected in L-edge XAS. In other words, we need to quantitatively relate L-edge XAS to ground- and excited-state electron configurations in order to extract charge and spin density changes beyond the notation of formal oxidation states. Such unified knowledge has broad impact on the mechanistic understanding of catalytic reactions because it directly answers the question “how charges and spins are spread in space” around redox-active metal sites.

In this study, we use a combination of state-of-the art experimental and computational methods, namely partial-fluorescence yield (PFY) detected XAS (PFY-XAS) on a liquid jet and quantum-chemical restricted active space (RAS) simulations, to render an improved description of L-edge XAS. With our approach we overcome what has been a challenge to date to quantitatively describe L-edge XAS on a consistent theoretical basis, including quantification of local charge and spin density changes for different formal oxidation states of the metal. L-edge XAS is affected by a number of factors;^21^,^32^ the structure of the complex,^24^,^26^ the covalency of the metal–ligand bonds,^19^,^20^,^33^ and the intra-atomic Coulomb and spin–orbit interactions in the 2p core and 3d valence shells.^26^,^34^–^36^ The *ab initio* RAS method used here includes all important interactions affecting the metal L-edge spectra^37^–^39^ and, being based on an explicit treatment of molecular orbitals, it offers the ability to directly relate local charge and spin densities with the L-edge spectrum. Redox and core-excitation processes can now be studied on an equal level of approximation, a prerequisite for unifying the corresponding pictures. Furthermore, by using an in-vacuum liquid jet with rapid replenishment of the sample we also overcome the severe challenge of soft X-ray induced sample damage to 3d-transition metal complexes.^22^,^40^

**Fig. 1:**
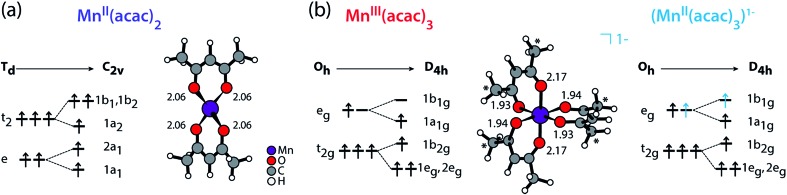
Schematic molecular orbital diagrams (ground-state electron configurations depicted with arrows) and structures (optimized with DFT functional B3LYP plus polarizable continuum model, Mn–O bond lengths in Å) of (a) Mn^II^(acac)_2_ in *T*_d_ and *C*_2v_ symmetry and of (b) Mn^III^(acac)_3_ and (Mn^II^(acac)_3_)^1–^ in *O*_h_ and *D*_4h_ symmetry. * in (b) mark methyl groups that were replaced with H in the restricted active space (RAS) X-ray spectrum calculations (with negligible effects on the spectra). Details on the structures can be found in the ESI.†

We compare the experimental L-edge XAS spectra of the two prototypical high-spin Mn complexes Mn^II^(acac)_2_ and Mn^III^(acac)_3_ measured in solution using the PFY mode to spectra calculated with the RAS approach. Mn^II^(acac)_2_ exhibits a nearly tetrahedral ligand environment with four Mn–O bonds, while Mn^III^(acac)_3_ is approximately an octahedral complex with six Mn–O bonds. The (acac)^–^ ligand has a formal charge of –1, which gives formal oxidation states of +2 (II) for the neutral Mn^II^(acac)_2_ complex and +3 (III) for the neutral Mn^III^(acac)_3_ complex. To separate the effects due to oxidation state changes from possible influences of variations in geometric structure, we introduce an artificial system, which exhibits a Mn^II^ oxidation state in the same geometry as Mn^III^acac_3_. Geometric structure, bonding and valence electronic structures of Mn^II^(acac)_2_, Mn^III^(acac)_3_ and (Mn^II^(acac)_3_)^1–^, are summarized in Fig. 1.

Our results show how the distinct changes in Mn L-edge XAS upon oxidation, especially the increase in incident energy with increasing Mn oxidation state, can be explained with the quantum-chemistry picture where the oxidation does not occur locally on the Mn site. We also discuss our findings in the context of the three most common explanations of the L-edge XAS shift to higher energies upon metal oxidation as discussed for Mn complexes by Glatzel *et al.*^32^ and van der Laan and Kirkman:^26^ First, the suspected decrease of core–hole screening with increasing oxidation state,^41^,^42^ second, the notion that changes in the relative number of 2p–3d (Q) and 3d–3d (U) direct Coulomb interactions shifts the L-edge XAS to higher energies by Q–U > 0,^26^ and, third, the speculation that for increasing oxidation states favorable 2p–3d exchange interactions decrease in the final core-excited states.^32^ It has remained unclear to date whether one or all of these concepts are necessary or sufficient to explain oxidation-state dependent L-edge XAS shifts. We make use here of the fact that RAS calculations for L-edge XAS now offer the unique possibility to study both the effects of oxidation or reduction and core excitation at an equal level. Understanding how changes in charge and spin densities around redox-active metal sites determine the metal L-edge absorption energies paves the route for a new understanding of catalytic reactions of 3d-transition metal complexes and metalloenzymes in general and is critical for studies specifically of the Mn cluster in photosystem II during the Kok cycle and related model complexes.^22^

## Materials and methods

### Experimental design

**Fig. 2:**
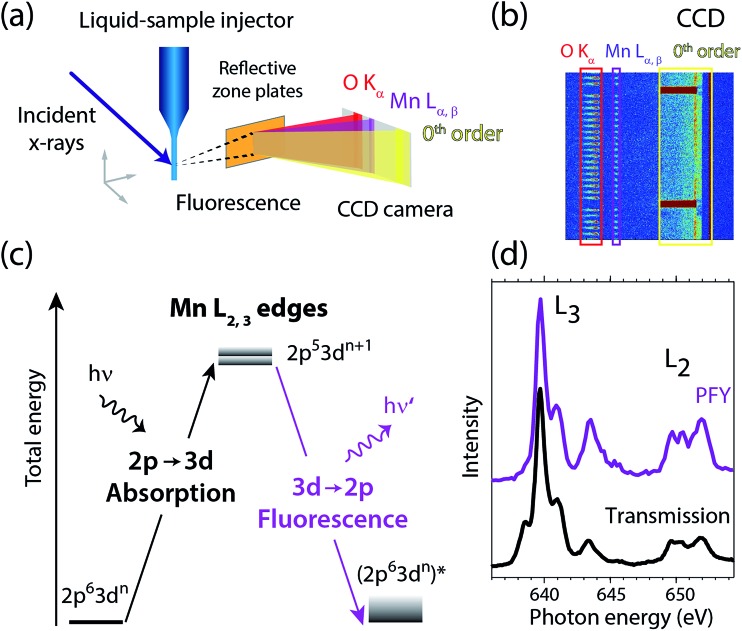
(a) Schematic depiction of the experimental setup for partial-fluorescence-yield X-ray absorption spectroscopy (PFY-XAS) with an in-vacuum liquid jet, a dispersive reflection-zone-plate (RZP) spectrometer, and a CCD camera. (b) Representative CCD image taken at a photon energy of 641.6 eV on a 100 mM Mn^III^(acac)_3_ solution sample as accumulated for 10 seconds (areas with Mn L_α,β_ and O K_α_ fluorescence and zeroth-order reflection are indicated). (c) Scheme of the transitions in Mn L-edge PFY-XAS in an approximate atomic-orbital picture with 2p–3d absorption from the initial ground state to the final core-excited states and 3d–2p (Mn L_α,β_) fluorescence decay. (d) Mn L-edge absorption spectra for Mn^2+^ in aqueous solution from MnCl_2_ in water taken with PFY-XAS (purple, taken from ref. 45) and in transmission (black, taken from ref. 43).

Fig. 2 summarizes our experimental approach to Mn L-edge XAS of dilute samples in solution. The schematic illustration of the experimental setup in Fig. 2(a) shows the essential components with the incident X-ray beam from the BESSY II synchrotron radiation facility, the in-vacuum liquid-sample injector, the reflective zone plate (RZP) as a dispersive element for spatial separation of Mn and O fluorescence and the CCD camera (Andor iKon-L) for fluorescence detection. The RZP spectrometer represents an improved version of our previous setup^43^ which we also used at the Linac Coherent Light Source (LCLS) X-ray free-electron laser.^22^,^44^,^45^ X-ray fluorescence from the sample mainly consists of Mn L_α,β_ emission at ∼640 eV from the solute and of O K_α_ at ∼525 eV from the solvent. Dispersion with our RZP spectrometer, optimized for high transmission,^46^ is essential for separating the weak Mn L_α,β_ signal of the dilute Mn samples from the overwhelming O K_α_ background. The energy of the photons incident on the liquid sample is scanned stepwise over the Mn L absorption edge, where at each step the Mn L_α,β_ partial fluorescence is detected. This corresponds to partial-fluorescence yield (PFY) detected X-ray absorption spectroscopy (PFY-XAS).^47^,^48^ With the example CCD image in Fig. 2(b), we demonstrate the excellent spatial separation of Mn and O fluorescence signals which is required for a good signal to noise ratio in the Mn L-edge PFY-XAS spectra. In Fig. 2(b), we also show the total fluorescence signal on the CCD, as reflected in the zeroth order of the RZP. For samples with sufficiently low concentrations, this is proportional to the incident flux and we use it for normalization of the spectra to the incident flux. Being interested in the bulk properties of the liquid samples, with this setup we overcome the difficulties imposed by surface-sensitive electron-yield (TEY) detected XAS. We employ a free-flowing liquid jet for continuous sample replenishment. At sufficient jet speed (see below), this approach avoids the risk of accumulating X-ray induced sample damage, which is known from L-edge XAS studies of radiation sensitive 3d transitions metals on solid samples^40^ or experiments that use liquid sample cells, based on X-ray transmissive membranes.^49^–^52^ Free-flowing liquid flatjets have also been used for soft X-ray absorption spectroscopy in transmission mode.^53^,^54^ For the present sample concentrations we determined in separate test experiments that spectral distortions due to reabsorption and inverse partial fluorescence yield effects are below the experimental noise level.

In a many-electron total-energy picture based on atomic orbitals as shown in Fig. 2(c), Mn L-edge XAS corresponds to the absorption of soft X-ray photons by Mn with transitions from the initial ground state Mn 2p^6^3d^*n*^ (where *n* is the number of Mn 3d electrons in a given oxidation state) to the final core-excited states Mn 2p^5^3d^*n*+1^. This corresponds to the promotion of a Mn 2p electron to empty or partially occupied Mn 3d-derived molecular orbitals (MOs) and, due to the dipole selection rule for 2p–3d transitions, L-edge XAS probes the d character of these MOs. The Mn L_α,β_ fluorescence that is detected originates from radiative decay of the core-excited XAS final states. State-dependent fluorescence yields for these decay transitions^47^,^55^,^56^ are the origin for deviations of the relative feature intensities in PFY-XAS spectra as compared to those in XAS as measured, *e.g.*, in transmission. In Fig. 2(d) we illustrate these deviations for Mn L-edge XAS of an aqueous MnCl_2_ solution. State-dependent fluorescence yields thus have to be taken into account in theoretical calculations aiming at reproducing the experimental PFY-XAS spectra.

### Partial-fluorescence-yield X-ray absorption spectroscopy (PFY-XAS) measurements

Mn L-edge PFY-XAS spectra were collected at the soft X-ray beamline U49-2_PGM1 at the synchrotron radiation facility BESSY II of the Helmholtz-Zentrum Berlin (Germany). The new RZP spectrometer and the in-vacuum liquid jet injector were installed in the previously described setup for fluorescence and resonant inelastic X-ray scattering (RIXS) investigations on liquid samples.^57^ The beamline flux at 640 eV was 5.4 × 10^12^ photons per s (with topping up mode of the electron storage ring). The size of the X-ray focus was 100 × 90 μm^2^ (horizontal × vertical), as observed with an Infinity K2 microscope on a YAG screen in the X-ray probing region and the beamline-monochromator slit was chosen to 90 μm corresponding to a bandwidth of the incident photon-energy around 300 MeV (Gaussian FWHM). The incident-energy step size was 100 MeV and CCD images were integrated for 10 seconds per step. The spectra of Mn^II^(acac)_2_ and Mn^III^(acac)_3_ were accumulated for 3.0 and 1.8 hours, respectively. The incident photon energy was calibrated with the Mn L-edge PFY-XAS spectrum of (Mn^II^(H_2_O)_6_)^2+^ from MnCl_2_ in a water jet (not shown), measured during the same beamtime with respect to the calibrated spectrum previously published in ref. 43. For this calibration the maximum of the L_3_ edge of (Mn^II^(H_2_O)_6_)^2+^ is located at 639.7 eV.

### Sample preparation

The solution samples were prepared as (i) ∼50 mM Mn^II^(acac)_2_ in absolute ethanol and (ii) ∼100 mM Mn^III^(acac)_3_ in acetylacetone. Calculated Mn L-edge absorption spectra of Mn^III^(acac)_3_ in acetylacetone and ethanol demonstrate that the solvent effect on the spectra is negligible.^54^ Possible differences of or induced by the different solvents will therefore not be further considered here. Samples and solvents were purchased from Sigma Aldrich and were used without further purification. Solution samples were delivered in an in-vacuum liquid jet, pumped from a 10 ml sample loop by a HPLC pump (JASCO PU-2085) and injected with a gas dynamic virtual nozzle injector^58^ at flow rates around 9 μl min^–1^ and 12 μl min^–1^ for the Mn^II^(acac)_2_ and Mn^III^(acac)_3_ samples, respectively. The sheath gas was N_2_ with a pressure of 10 bar. The jet diameter was determined to be 20 ± 4 μm and 22 ± 4 μm for the Mn^II^(acac)_2_ and Mn^III^(acac)_3_ solution samples, respectively, as observed with an Infinity K2 microscope. For the Mn^II^(acac)_2_ and Mn^III^(acac)_3_ solution samples this corresponds to a vertical sample speed of 0.45 ± 0.20 m s^–1^ and 0.53 ± 0.20 m s^–1^ through the X-ray interaction region and X-ray exposure times of 200 ± 90 μs and 170 ± 70 μs, respectively.

### *Ab initio* restricted active space (RAS) calculations of Mn L-edge XAS

Calculations of molecular systems were performed using ground state geometries optimized in solution with the density-functional theory (DFT) hybrid functional B3LYP^59^ and the 6-31G(d) basis set (see Fig. 1 for calculated structures, bonding and valence electronic structures, Fig. S1† for orbital shapes and the ESI† for further details of the calculations). To minimize computational cost, the Mn^III^(acac)_3_ calculations were performed on a truncated complex where six methyl groups were replaced by hydrogen atoms (Fig. 1(b)). We checked that this did not notably change the spectrum. All calculations were performed in a solvent environment approximated by a polarized continuum model (PCM).^60^

The RAS calculations of the Mn L-edge spectra were performed with MOLCAS 7.9.^61^ The five Mn 3d-derived orbitals were placed in the RAS2 space where all possible excitations were allowed. The Mn 2p orbitals were placed in the RAS3 space, allowing a maximum of five electrons, *i.e.*, at least one hole. To ensure that the hole stayed in the 2p instead of the higher-lying 3p orbitals, the former had to be frozen in the RASSCF optimizations of the core-excited states. The RASSCF wavefunction optimizations were performed using the state average formalism, which means that the same orbitals were used for all states of a specific spin and symmetry.^62^ In the calculations of the Mn L-edge PFY-XAS spectra, all possible configurations that represent valence excitations and single core excitations were included.

Interactions between electrons outside the active space, including all ligand-dominated orbitals, are treated at the level of second-order perturbation theory (RASPT2) using the multi-state formalism.^63^ Scalar relativistic effects were included by using a Douglas–Kroll Hamiltonian in combination with a relativistic atomic natural orbital basis set of triple-zeta quality, ANO-RCC-VTZP, for the PFY-XAS calculations, and double-zeta quality, ANO-RCC-VDZP for XAS cross section.^64^,^65^ The oscillator strengths (absorption strengths) were calculated between orthogonal states formed from a RAS state-interaction approach that also includes spin–orbit coupling.^66^ UV-Vis spectra of Mn^III^(acac)_3_ show metal-centered ligand-field excitations between 2.1 and 2.4 eV.^67^–^69^ Our RAS excitation energies of 2.4 eV and 2.6 eV roughly agree with those values showing the accuracy of these calculations.

The Mn L-edge PFY-XAS spectra were calculated by integrating the emission intensity of 2p–3d RIXS spectra: for each incident photon energy the transition matrix element was calculated for all allowed L-edge excitations and for all possible fluorescence decay channels within the active space. The PFY-XAS spectra were obtained from the absorption probabilities, weighted by the sum of transition matrix elements for the decay channels. The Lorentzian (lifetime) broadening was (HWHM) 0.2 eV and 0.7 eV for the L_3_ and L_2_ absorption edges, respectively.^70^ The monochromator bandwidth is simulated with an additional Gaussian broadening of 0.3 eV (FWHM). To align the calculated spectra with the experimental PFY-XAS spectra, the calculated intensities were normalized to one at maximum and the incident energies were shifted by –3.09 eV to align with the measured Mn^II^(acac)_2_ spectrum at the L_3_-edge maxima. The relative photon energies of Mn^II^(acac)_2_ and Mn^III^(acac)_3_ spectra are displayed as calculated. The same applies for the calculated Mn L-edge XAS spectra, which were shifted by –4.94 eV. The different shifts applied to PFY-XAS and XAS spectra account for differences inherent to calculations with different basis sets.

The electronic structures of the two complexes were analyzed by calculating Mulliken spin populations and LoProp charges,^71^ as well as charge and spin densities using the MultiWfn package.^71^,^72^ An interpretation of the spectrum in terms of orbital excitations was made by calculating differences in natural occupation numbers for the active orbitals associated with each transition.^38^

## Results and discussion

### Measured and calculated Mn L-edge XAS spectra of Mn^II^(acac)_2_ and Mn^III^(acac)_3_

**Fig. 3:**
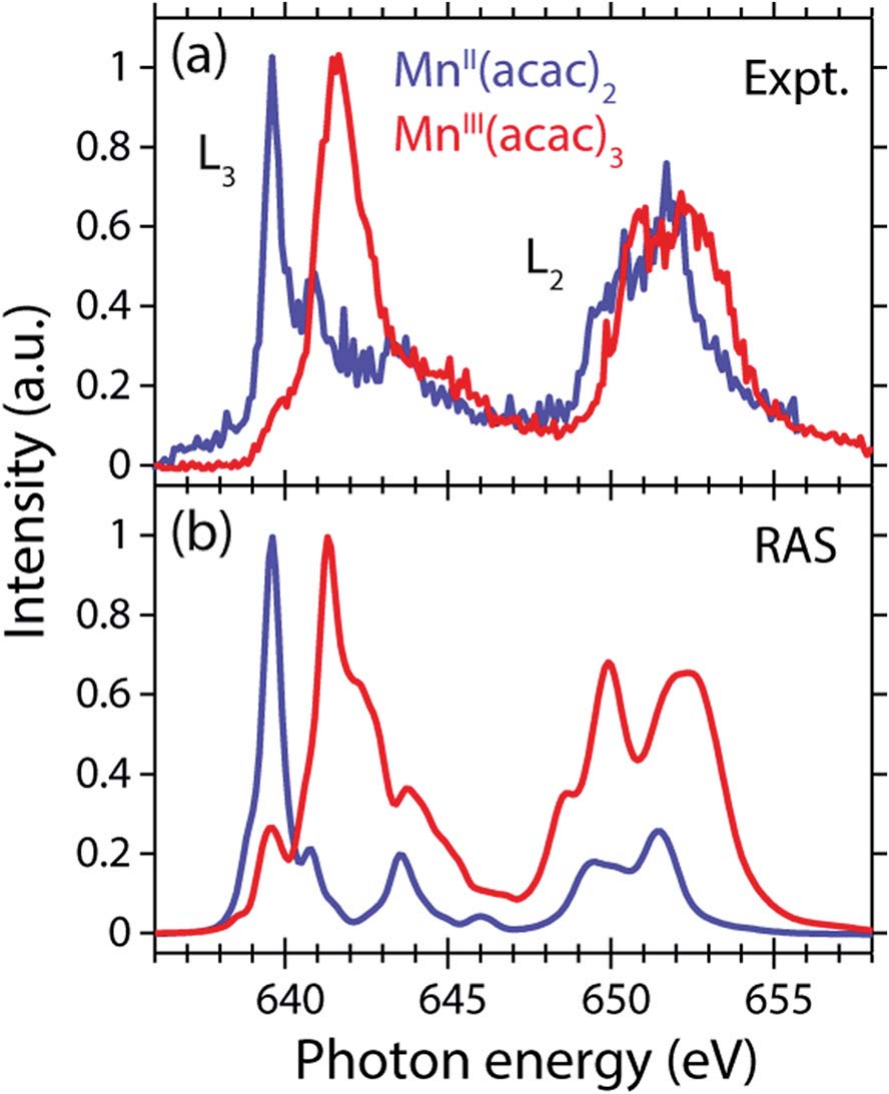
Comparison of (a) measured and (b) calculated partial-fluorescence-yield (PFY) Mn L-edge absorption spectra of Mn^II^(acac)_2_ and Mn^III^(acac)_3_. (a) Experimental spectra of ∼0.05 mol l^–1^ Mn^II^(acac)_2_ solution in ethanol and ∼0.1 mol l^–1^ Mn^III^(acac)_3_ solution in acetylacetone. (b) Calculated spectra from the restricted active space (RAS) calculations with photon energies of the Mn^II^(acac)_2_ spectrum shifted to match the experimental spectrum at the L_3_-edge maxima, while keeping the relative photon energies of Mn^II^(acac)_2_ and Mn^III^(acac)_3_ spectra as calculated. In all spectra the intensities were normalized to one at maximum.

The measured and calculated Mn L-edge PFY-XAS spectra of Mn^II^(acac)_2_ and Mn^III^(acac)_3_ in solution are shown in Fig. 3. The Mn^II^(acac)_2_ spectrum has a narrow main peak at the L_3_ edge at 639.6 eV and two peaks at 641 eV and 643.5 eV on a somewhat broad intensity distribution extending up to 645 eV, which is characteristic of ionic Mn^II^ systems.^25^,^43^,^73^ The L_3_ edge of Mn^III^(acac)_3_ in contrast exhibits a broad main peak at 641.6 eV with shoulders at 639.5 and 645 eV. For both systems the L_2_ edge is comparably broad with two maxima or shoulders separated by around 2 eV.

Our solution Mn^II^(acac)_2_ spectrum is very similar to the solid-state spectrum measured with total electron yield (TEY) XAS in ref. 73 while our solution Mn^III^(acac)_3_ spectrum is strikingly different from that in the solid state. The first observation shows that the effect of solvent environment on the spectrum of Mn^II^(acac)_2_ is relatively small. This is consistent with observations based on the K-edge spectra of iron cyanides in solution,^17^ and we checked with additional RAS simulations that the influence of solvent effects on the L-edge XAS spectra for the systems studied here are very small. We assign the difference between our solution spectrum and the solid-state spectra of Mn^III^(acac)_3_ to X-ray induced sample damage in the solid-state measurements.^32^,^73^ With our experimental parameters, we estimate the maximum doses accumulated in a probed sample volume to be 5.2 ± 2.3 kGy and 4.6 ± 1.7 kGy (1 Gy = 1 J kg^–1^) for the Mn^II^(acac)_2_ and Mn^III^(acac)_3_ solution samples, respectively (see the ESI† for details). This is safely below 10^6^ Gy,^74^ the typical order of magnitude that is relevant for X-ray induced sample damage.^40^,^74^ This makes the measured spectra in Fig. 3(a) amenable for unambiguous interpretation of the effects of changes in oxidation state.

The largest difference between the spectra of the two complexes is the shift of the L_3_-edge maximum by 2.0 eV to higher energies when going from Mn^II^(acac)_2_ to Mn^III^(acac)_3_. Quantifying the changes with the first moment of the L_3_ edge or the first moment of the whole L edge (L_3_ + L_2_) gives smaller energy shifts (Table 1). In this study, we focus on the L_3_-edge maximum as it is most accurately defined and least affected by state-dependent fluorescence yield, which facilitates comparisons to other complexes and previous results in literature. The shift roughly agrees with the oxidation-state dependent L-edge shift of 1.6 ± 0.3 eV, observed for the L_3_-edge maxima of other high-spin Mn complexes with oxidation states ranging from Mn^II^ to Mn^III^ and Mn^IV^, as reported in ref. 22 as well as for Mn compounds in ref. 13, 25, 27 and 75.

**Table 1:** Measured and calculated energies in Mn L-edge partial-fluorescence yield (PFY)-XAS and XAS spectra of Mn^II^(acac)_2_, Mn^III^(acac)_3_, and (Mn^II^(acac)_3_)^1–^ (in eV)

Complex	Method	L_3_ Edge maximum	L_3_ 1^st^ moment^*a*^	L_2_ 1^st^ moment	L_3_ + L_2_ 1^st^ moment
Mn^II^(acac)_2_	Experiment PFY-XAS	639.6	641.9	651.5	646.8
	**Theory RAS**				
	PFY-XAS^*b*^	639.6	640.7	650.9	644.3
	XAS^*b*^	639.6	640.4	650.9	642.7
	Sextet XAS^*b*^	639.6	639.6	650.5	641.4
Mn^III^(acac)_3_	Experiment PFY-XAS	641.6	642.8	652.4	647.6
	**Theory RAS**				
	PFY-XAS^*c*^	641.3	642.3	651.3	647.1
	XAS^*c*^	641.3	641.6	651.2	645.0
	Quintet XAS^*c*^	641.2	641.2	651.3	643.2
(Mn^II^(acac)_3_)^1–^	**Theory RAS**				
	XAS^*c*^	639.7	640.2	650.6	642.5
	Sextet XAS^*c*^	639.7	639.3	649.9	641.1

^*a*^The 1^st^ moment is the center of gravity energy (sum of all energies times intensities divided by the sum of all intensities).

^*b*^Aligned to L_3_-edge maximum of experiment.

^*c*^Shifted by same amount as Mn^II^(acac)_2_ RAS spectra.

The calculated *ab initio* RAS spectra of Mn^II^(acac)_2_ and Mn^III^(acac)_3_ are shown in Fig. 3(b). The overall agreement with experiment is good with well-reproduced multiplet structures in terms of relative energies. The RAS spectrum of Mn^II^(acac)_2_ is too low in intensity at the high-energy side of the L_3_ edge at 641–645 eV and generally at the L_2_ edge. For Mn^III^(acac)_3_ the RAS spectrum is too intense in the first peak/shoulder at 639.5 eV, at the high-energy side of the L_3_ edge at 643–647 eV and at the low-energy side of the L_2_ edge.

The deviations between experiment and theory on the high-energy side of the L_3_ edge of Mn^II^(acac)_2_ may be related to the deficiencies of RAS in accounting for charge transfer (CT) when using a minimal active space consisting of only the five metal-dominated orbitals.^19^,^76^ In our RAS calculations, CT satellites are missing because ligand-dominated orbitals are not included in the active space and hence core-excited states with dominant LMCT character cannot be reached. However, the minimal active space used here guarantees that all systems are treated at an equal basis making it more straightforward to assign spectral changes and energy shifts.

The deviations in the first peak of Mn^III^(acac)_3_ and in the L_2_ edge of both systems can be explained by an incomplete description of PFY-XAS in contrast to the XAS cross section, because these regions are most sensitive to state-dependent fluorescence yield effects.^47^,^55^,^56^ The L_3_-edge maximum is largely independent of the detection mode, which makes it an appropriate observable when comparing theory and experiment.

The calculated shift of 1.7 eV between Mn^II^(acac)_2_ and Mn^III^(acac)_3_ agrees with the experimental observation to within 0.3 eV (the RAS spectra were both shifted by the same amount of –3.09 eV to match calculated and measured L_3_-edge maximum energies of Mn^II^(acac)_2_). The agreement of calculated and measured shifts indicates that RAS calculations, which are independent of any adjustable parameters, can be used to predict the incident-photon energy shifts in the L-edge absorption spectra of 3d transition-metal complexes upon changing their nominal oxidation state. RAS calculations of iron K pre-edges show average deviations of 0.2 eV when predicting the energy shift between complexes with similar ligand environments and active space selections.^77^

### Decomposition of the spectra into spin multiplet components

**Fig. 4:**
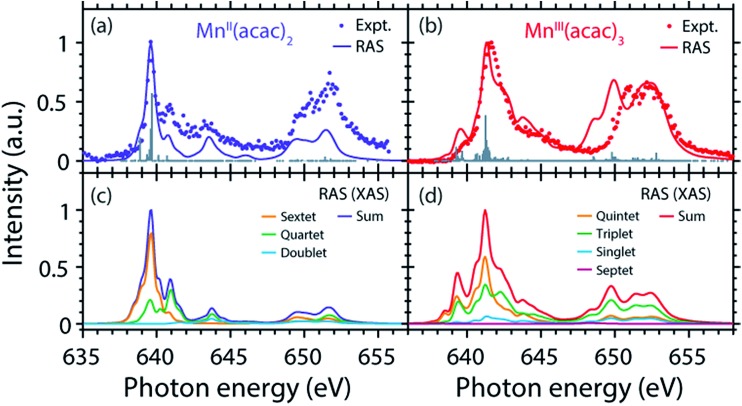
(a and b) Calculated RAS and experimental PFY-XAS spectra of (a) Mn^II^(acac)_2_ and (b) Mn^III^(acac)_3_ (note the difference in the plotted energy ranges for Mn^II^(acac)_2_ and Mn^III^(acac)_3_). (c and d) Calculated absorption spectra (XAS not PFY) decomposed into the relative contributions of the (spin) multiplicities in LS- or Russel–Saunders coupling in the final core-excited states for (c) Mn^II^(acac)_2_ and (d) Mn^III^(acac)_3_.

The agreement of the experimental and RAS spectra in terms of shapes and relative energy shift motivates analyzing these observables in more detail. We focus on the question to what extent they can be correlated with differences in the oxidation state or other differences in ground or final state properties of Mn^II^(acac)_2_ and Mn^III^(acac)_3_. Following earlier studies,^38^,^39^,^75^ the calculated RAS spectra of Mn^II^(acac)_2_ and Mn^III^(acac)_3_ were decomposed into final-state spin multiplicities as shown in Fig. 4. This decomposition was done for XAS instead of the PFY-XAS spectra due to our current limitations in performing these analyses for PFY-XAS. The comparison of calculated XAS and PFY-XAS spectra shows that the multiplet structures are identical in terms of relative transition energies within the spectral bandwidth considered here (compare Fig. 4(a) with 4(c) and 4(b) with 4(d)), hence justifying this approximation.

We consider angular-momentum coupling in the core-excited states within the Russel–Saunders or LS-coupling scheme (spin multiplicity 2S + 1 with the spin angular momentum S).^35^,^78^ LS coupling is the only scheme for which we can currently extract angular momenta in our RAS calculations. While LS coupling is a very good approximation for the initial ground state Mn 3d^*n*^ configurations,^78^,^79^ it is known to fail for the Mn 2p^5^3d^*n*+1^ core-excited state configurations because the 2p spin–orbit interaction is of comparable magnitude as the Coulomb interactions.^26^,^36^,^79^ It is still informative to decompose the final states in LS coupling as this enables a conceptual understanding of the L_3_-edge multiplet features and their energies.

Because of the strong 2p spin–orbit coupling in the final core-excited states the total spin S is not strictly preserved in the 2p–3d excitation process for both Mn^II^(acac)_2_ (sextet e^2^t_2_^3^ ground state in *T*_d_ symmetry) and Mn^III^(acac)_3_ (quintet t_2g_^3^e_g_^1^ ground state in *O*_h_ symmetry). Indeed for both systems we find corresponding spectral contributions with considerable intensities for sextet (Δ*S* = 0), quartet (Δ*S* = –1), and doublet (Δ*S* = –2) multiplicities for Mn^II^(acac)_2_ and quintet (Δ*S* = 0), triplet (Δ*S* = –1), and singlet (Δ*S* = –2) for Mn^III^(acac)_3_ (Fig. 4, note that septet states with Δ*S* = +1 are possible for Mn^III^ but their intensity is close to zero^35^). Contributions with the same spin multiplicities as in the ground states (Δ*S* = 0) are strongest for both systems, in particular in the L_3_-edge maximum.

The average energies of the multiplicity components increase with increasing Δ*S* from 0 to –1 and –2 and this explains to some extent the experimentally observed shapes of the Mn^II^ and Mn^III^ spectra (see *e.g.* the peak at 641 eV in the Mn^II^(acac)_2_ spectrum originating from quartet, Δ*S* = –1, components).

This shows that for the high-spin complexes studied here with comparably weak ligand fields, the spectral shape is mainly determined by differences in electron–electron repulsion in the final core-excited states instead of differences in orbital energies (see Fig. S2 in the ESI†). In other words, local intra-atomic (multiplet) effects in the final core-excited states seem to dominate the spectrum rather than inter-atomic or molecular (ligand-field) effects.

Nevertheless, the relative L-edge XAS shift of the Mn^II^(acac)_2_ and Mn^III^(acac)_3_ spectra remains elusive. We therefore turn to an analysis of calculated charge and spin density distributions and we start by analyzing the initial ground states of the systems.

### Radial charge density (RCD) and radial spin density (RSD) from RAS in the initial ground states

The local charge assigned to Mn according to our RASSCF calculations (LoProp, see Materials and methods) are 1.56 for Mn^II^(acac)_2_ and 2.08 for Mn^III^(acac)_3_ as given in Table 2. This does not match the formal oxidation states of 2+ and 3+, and the difference between the two complexes is not unity as expected from a one-electron oxidation. DFT/B3LYP calculations give even smaller values, namely 1.37 and 1.61 for Mn^II^(acac)_2_ and Mn^III^(acac)_3_, respectively. The weak correlation between charge (or local effective charge) and formal oxidation state agrees with many previous observations^29^–^31^,^80^ which also show that metal charges are strongly dependent on the ligand. For many 3d transition-metal systems the formal oxidation state is better reflected by the spin population on the metal because it is relatively insensitive to the nature of the ligand.^30^ For Mn^II^(acac)_2_ and Mn^III^(acac)_3_ the RAS Mulliken^81^ spin populations for Mn indeed are 4.93 and 3.85, close to the number of unpaired electrons (5 and 4 electrons) for the two systems (see Table 2). We thus find, in agreement with Blomberg and Siegbahn,^30^ that in quantum chemistry calculations the spin populations, rather than the charges, are good fingerprints for the formal oxidation state.

**Table 2:** Charge populations (in units of one electron) and spin populations (number of valence electrons of the same spin) at the Mn center in the electronic ground states of Mn^II^(acac)_2_ and Mn^III^(acac)_3_ and (Mn^II^(acac)_3_)^1–^ calculated using restricted active space self-consistent field (RASSCF) calculations and DFT calculations with the B3LYP functional

Complex	Formal charge	Charge LoProp RASSCF	Charge LoProp B3LYP	Unpaired electrons	Spin Mulliken RASSCF	Spin Mulliken B3LYP
Mn^III^(acac)_3_	3+	2.08+	1.61+	4	3.85	3.90
Mn^II^(acac)_2_	2+	1.56+	1.37+	5	4.93	4.81
(Mn^II^(acac)_3_)^1–^	2+	1.52+	1.37+	5	4.92	4.82

The important scientific question is how the experimental observables, the differences in spectral shape and the energy shift of the L-edge, reflect these changes in local charge and spin density as a function of the formal oxidation state. Apparently, a molecular-orbital based method that in addition accurately describes the L-edge XAS process is needed to answer this question. For the metal complexes studied here the good agreement between RAS calculations and experiment makes it possible, for the first time to the best of our knowledge, to analyze the changes in charge and spin densities of the metal in different oxidation states.

For this and as previously done by Johansson *et al.*,^29^ we spatially resolve the radial charge density (RCD) and the radial spin density distributions (RSD). We start by comparing the artificial system (Mn^II^(acac)_3_)^1–^ (with geometry fixed as in Mn^III^(acac)_3_, see Methods and materials section) with Mn^III^(acac)_3_ to safely exclude possible influences by differences in geometry. Our spin and charge population analysis in Table 2, however, shows that the values for (Mn^II^(acac)_3_)^1–^ agree within 0.04 with the corresponding values of Mn^II^(acac)_2_, which indicates that local charge and spin densities are independent from the influence of the Mn–O ligand bonding geometry and that the (Mn^II^(acac)_3_)^1–^ to Mn^III^(acac)_3_ comparison is a valid starting point for comparing the charge and spin densities in Mn^II^(acac)_2_ to Mn^III^(acac)_3_.

**Fig. 5:**
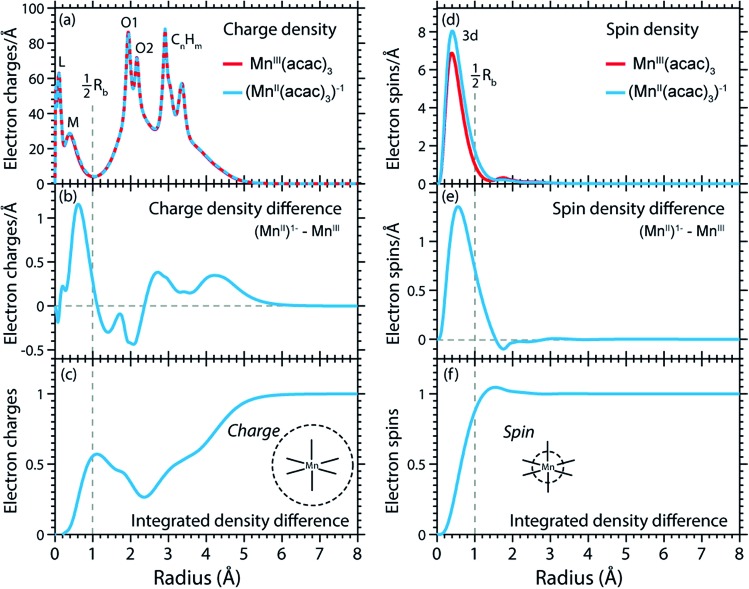
(a) Radial charge density (RCD) in the ground states of Mn^III^(acac)_3_ and (Mn^II^(acac)_3_)^–1^ (same geometry as Mn^III^(acac)_3_ plus one additional electron). (b) RCD difference (RCD of (Mn^II^(acac)_3_)^–1^ from (a) minus RCD of Mn^III^(acac)_3_ from (a)). (c) Integrated RCD difference. (d) Radial spin density (RSD) of Mn^III^(acac)_3_ and (Mn^II^(acac)_3_)^–1^. (e) RSD difference (RSD of (Mn^II^(acac)_3_)^–1^ from (d) minus RSD of Mn^III^(acac)_3_ from (d)). (f) Integrated RSD difference. All properties were extracted from RAS calculations and are plotted *versus* the radius of a sphere around Mn. The dashed vertical lines approximately indicate half the Mn–O bond length *R*_b_. Note that some methyl groups were replaced by H atoms (see ESI†) with negligible effect on the calculated charge and spin density distributions.

The calculated RCD and RSD are plotted for the ground states of Mn^III^(acac)_3_ and (Mn^II^(acac)_3_)^1–^ in Fig. 5 ((Mn^II^(acac)_3_)^1–^ and Mn^III^(acac)_3_ are abbreviated as (Mn^II^)^1–^ and Mn^III^). The data were extracted from our RAS calculations by placing a spherical shell of radius r and thickness dr around Mn, with volume d*V*(*r*) = 4π*r*^2^d*r*, and plotting the average electron charge and spin densities in this spherical shell *versus r*. The RCD and the RSD are α + β and α – β radial electron densities *ρ*_α+β_(*r*) and *ρ*_α–β_(*r*), respectively, with α spin up and β spin down. The units are electron charges per Å and electron spins per Å (solid-angle integrated radial densities). With the RCD and RSD differences, we then evaluate charge and spin density differences between Mn^III^(acac)_3_ and (Mn^II^(acac)_3_)^1–^. With the integrated RCD and RSD differences as a function of the radius *R* (integrals over *ρ*_α+β_(*r*) × 4π*r*^2^d*r* and *ρ*_α–β_(*r*) × 4π*r*^2^d*r* from *r* = 0 to *r* = *R*), finally, we quantify the amount of electron charges and spins contained in a sphere of radius *R* around Mn in units of electron charges and electron spins.

In the RCDs in Fig. 5(a), peaks corresponding to the atomic Mn L-shell (main quantum number 2) at 0.1 Å, and M-shell (main quantum number 3) at 0.4 Å are visible, as well as peaks corresponding to ligating O atoms at 2 Å and other atoms in the (acac)^–^ ligands (>2.5 Å). A dashed line marks the half Mn–O bond length at 1 Å. The RCD difference in Fig. 5(b) and its integral in Fig. 5(c) clearly shows that the additional charge in (Mn^II^(acac)_3_)^1–^ compared to Mn^III^(acac)_3_ leads to a redistribution of charge over the whole molecule with considerable amplitude out to 6 Å. Electron charge accumulates on the outside of the M shell, is depleted from the Mn–O bond region as well as at the ligating oxygen atoms, and accumulates on the (acac)^–^ ligands. With the integrated RCD in Fig. 5(c), we infer that +0.55 electron charges are located within 1 Å of Mn, and that there is a depletion of the Mn–O bonds of –0.3 charges while +0.75 charges accumulate on the (acac)^–^ ligands. The sum of all changes at 5–6 Å is evidently equal to 1.

The RSDs of Mn^III^(acac)_3_ and (Mn^II^(acac)_3_)^1–^ in Fig. 5(d) draw a dramatically different picture. The entire electron spin density in both systems is concentrated at distances below 1 Å with a peak at 0.4 Å (Fig. 5(e)), the location of the 3d shell (in contrast to the labeling in the RCDs, we label the peak at 0.4 Å in the RSDs as the Mn 3d-shell density).

We can explain this dramatic imbalance of charge and spin density distributions following the reasoning of Johansson *et al.*^29^ It is important to note that while the RCDs consider all electrons (*e.g.* the sum of the 3s, 3p and 3d electrons for the M-shell), the RSDs represent the electron density solely of the unpaired electrons in the singly occupied orbitals (*e.g.* of all M-shell electrons only the contribution due to the 3d-shell is considered). The changes in the RSD therefore reflect the added (spin-up) electron, and spin density localizes at the Mn 3d shell due to favorable exchange Coulomb interactions with the other partially occupied orbitals. This principle is equivalent to the first of Hund's rules. We note an expansion of the Mn 3d shell by 0.01 to 0.02 Å upon reduction of the Mn^III^ complex (Fig. 5(d)). We explain this observation with the “accommodation” of the extra electron in the 3d shell, while minimizing electron–electron repulsion due to direct Coulomb interaction. As the electron is added, polarization effects of the electrons in the doubly occupied orbitals delocalize charge over the whole molecule to minimize the direct Coulomb repulsion. The changes in the RCD upon reduction (see Fig. 5(b)) reflect the effect both on the electrons in the partially occupied 3d orbitals and in the doubly occupied orbitals of the complex. The latter are invisible in the RSDs but visible in the RCDs. The opposite effects on the charge and spin density distributions in an ensemble of electrons render charge and spin densities as “independent observables”.^29^ The results in Fig. 5 are consistent with analyses of other Mn complexes where it was concluded that oxidation-state changes do not occur on the Mn atoms but on the ligands,^31^ confirming that reduction or oxidation of the molecule is not a reduction or oxidation of the metal ion but of the whole molecule.^29^

### Uncoupling the L-edge XAS shift from influences of the ligand environment

**Fig. 6:**
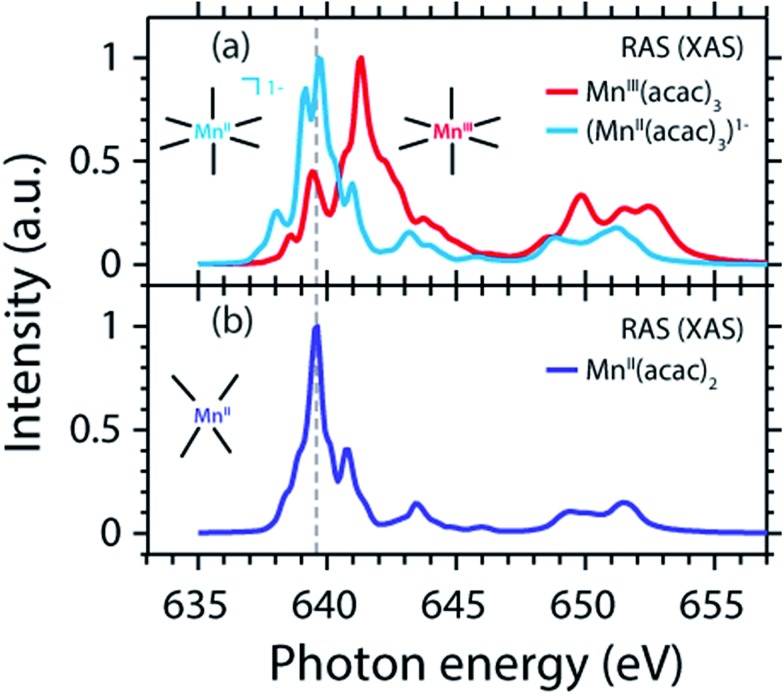
(a) Calculated Mn L-edge absorption spectrum of (Mn^II^(acac)_3_)^1–^ (same geometry as Mn^III^(acac)_3_ with one additional electron) compared to Mn^III^(acac)_3_ (same as in Fig. 4(d)). (b) Calculated spectrum of Mn^II^(acac)_2_ (same as in Fig. 4(c)). Relative energies of all spectra are displayed as calculated.

We proceed with establishing how the extra electron in the artificial (Mn^II^(acac)_3_)^1–^ complex is expressed in the XAS observable. The calculated RAS spectrum (XAS) of (Mn^II^(acac)_3_)^1–^ is compared to the Mn^III^(acac)_3_ spectrum in Fig. 6(a).

Adding an electron to Mn^III^(acac)_3_ without changing structure shifts the spectrum by –1.4 eV (L_3_-edge maximum). This agrees, within 0.2 eV, with the calculated Mn^II^(acac)_2_–Mn^III^(acac)_3_ shift (Table 1), which motivates finding a common explanation. Comparing spectra (XAS) of (Mn^II^(acac)_3_)^1–^ and Mn^II^(acac)_2_ evidences in particular that intensities are redistributed but the structures of the spectra and the L-edge absorption energies, in particular, are very similar. A largely constant value for the L_3_-edge energy with redistribution of intensities between the multiplet components for Mn ions in different ligand or crystal fields is consistent with the seminal multiplet calculations of de Groot^24^ and van der Laan.^26^ The remaining small L_3_-edge energy difference of 0.2 eV between (Mn^II^(acac)_3_)^1–^ and Mn^II^(acac)_2_ is also consistent with the shift of 0.1 eV for high-spin as well as for low-spin inorganic Fe complexes with the same formal oxidation state but different ligand fields.^19^ Importantly, we can conclude that the differences in ligand environment between Mn^II^(acac)_2_ and Mn^III^(acac)_3_ only have a minor effect on the L_3_-edge maximum shift (on the 1 eV level addressed here).

### Radial charge distribution (RCD) and radial spin distribution (RSD) from RAS in the final core-excited states

We can now transfer our analysis of radial charge and spin density distributions calculated in RAS from the initial ground states to the final core-excited XAS states. In our spectral spin multiplicity analyses for Mn^II^(acac)_2_, Mn^III^(acac)_3_ (Fig. 4) and (Mn^II^(acac)_3_)^1–^ we have identified the predominant contribution of final core-excited states with Δ*S* = 0 in the L_3_-edge (see Fig. S3 in the ESI† for the spin multiplicity analysis of the (Mn^II^(acac)_3_)^1–^ spectrum). To keep the analysis of our RAS calculations manageable, we average over five core-excited states with Δ*S* = 0, one for each of the five most intense transitions in the L_3_-edge. Analysis of the relative spin orientations shows that they are representative for the majority of the Δ*S* = 0 core-excited states.

**Fig. 7:**
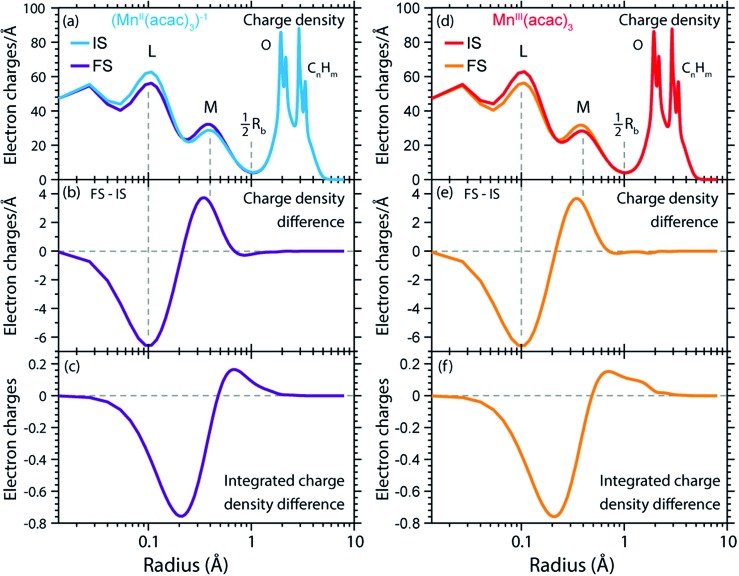
(a) Radial charge densities (RCD) from RAS of (Mn^II^(acac)_3_)^1–^ in the initial ground states (IS) and averaged over the final core-excited states (FS). (b) RCD difference of (Mn^II^(acac)_3_)^1–^ (RCD of final core-excited state minus RCD of initial ground state, FS-IS). (c) Integrated RCD difference of (Mn^II^(acac)_3_)^1–^. (d)–(f) The same for Mn^III^(acac)_3_. The dashed vertical lines indicate the location of L (2s, 2p) and M (3s, 3p, 3d) shell maxima and approximately half the Mn–O bond length *R*_b_.

The RCDs of initial ground and final core-excited states of (Mn^II^(acac)_3_)^1–^ and Mn^III^(acac)_3_ are compared in Fig. 7, where the latter are averaged over all final states and spin multiplicities. Upon 2p–3d excitation the changes in the RCDs are very similar in both systems. Note that these curves are shown on a logarithmic scale to focus on the intra-atomic density differences around Mn. Consistent with the expected 2p–3d excitation process, it is apparent from Fig. 7(a, b, d and e) that the charge density in the L-shell at 0.1 Å decreases, while it increases in the M-shell at 0.4 Å. For both systems we observe a small depletion of charge density at around the center of the Mn–O bond at 1 Å, reflecting a minimal polarization due to Coulomb repulsion to the charge density added in the M-shell upon 2p–3d core excitation. Above this distance, charge is not significantly reacting to the excitation process. Our observations are consistent with the notion by van der Laan *et al.* that in L-edge XAS the 2p “core electron can be excited into an efficient screening orbital so that the perturbation on the remaining ground-state electrons is small”.^82^ De Groot stated similarly that in L-edge XAS “because the 3d electrons are relatively localized, this is an almost self-screened process”.^76^ Our results thus visualize, validate and quantify fundamental notions of L-edge XAS of 3d transition-metal systems.

**Fig. 8:**
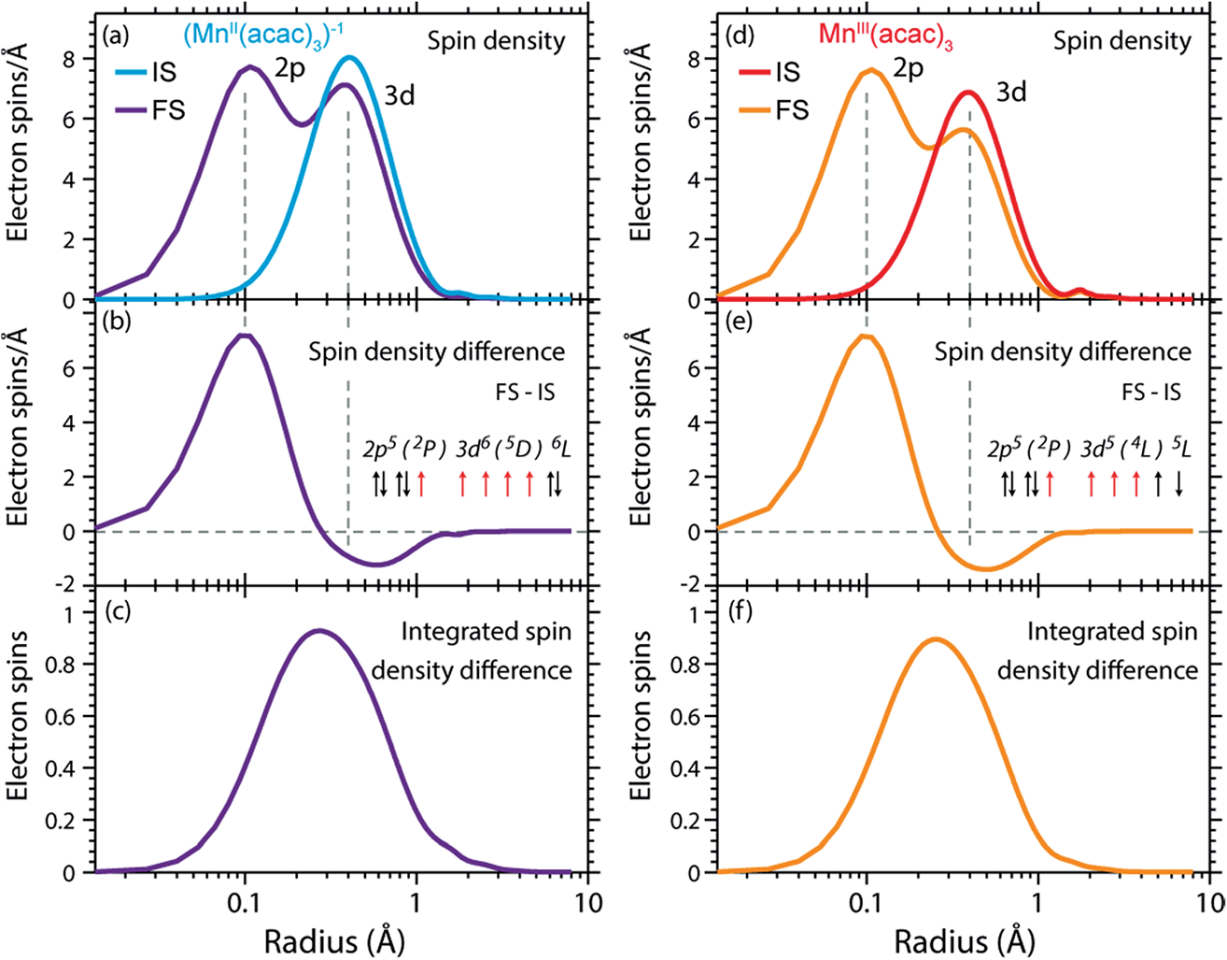
(a) Radial spin densities (RSD) from RAS of (Mn^II^(acac)_3_)^1–^ in the initial ground states (IS) and final core-excited states (FS), where the latter are averaged over five selected final core-excited states in the L_3_-edge with Δ*S* = 0 (quintet for Mn^III^ and sextet for Mn^II^, see main text). (b) RSD difference plot of (Mn^II^(acac)_3_)^1–^ (RSD of final core-excited states minus RSD of initial ground state, FS-IS). (c) Integrated RSD difference of (Mn^II^(acac)_3_)^1–^. (d)–(f) The same for Mn^III^(acac)_3_. The dashed vertical lines indicate the location of the 2p and 3d shell maxima. Insets in (b) and (e): schematic depictions of the dominant spin configurations in the final core-excited states (black: paired spins, red: unpaired spins, term symbols in LS-coupling, for initial-state and other final-state configurations see Table S1†).

The radial spin densities (RSDs) of (Mn^II^(acac)_3_)^1–^ and Mn^III^(acac)_3_ in the initial ground and final core-excited states are compared in Fig. 8. For both (Mn^II^(acac)_3_)^1–^ and Mn^III^(acac)_3_ they show electron spin density increase in the 2p shell at 0.1 Å and a decrease in the 3d shell at 0.4 Å upon 2p–3d excitation. The integrated RSDs (Fig. 8(c and f)) show that for both systems the 2p spin density increases by one and the 3d spin density decreases by one upon 2p–3d excitation. This important observation allows us to determine the dominant 2p and 3d spin configurations (one-electron spin orientations in LS-coupling) in the final core-excited states of the two systems. For sextet Mn^II^ (Δ*S* = 0) there is only one possible spin configuration (see Table S1 in the ESI†) with parallel 2p and 3d spins (spin orientation of the unpaired 2p electron in 2p^5^ relative to that of the 3d^*n*+1^ net spin). For quintet Mn^III^ (Δ*S* = 0), in contrast, two configurations are possible with either parallel or antiparallel 2p and 3d spins and with different numbers of effectively unpaired 3d electrons (Table S1†). The Mn^III^ RSDs and their (integrated) difference in Fig. 8(d–f) clearly identify the predominant spin configuration in the Mn^III^ quintet final states (Δ*S* = 0) as the one with parallel 2p and 3d spins. For final states with antiparallel 2p and 3d spins the 2p and 3d maxima in the RSDs in Fig. 8(d) and their difference would have opposite signs. For mixed final states with similar contributions of parallel and antiparallel 2p and 3d spins the changes in the spin densities would be less than one. We thus find that the preference for parallel 2p and 3d spin orientation is kept despite the large 2p spin–orbit interaction and hence the nominal inapplicability of LS coupling.

On a more detailed level, we observe a contraction of the 3d shell by 0.02–0.03 Å after 2p–3d core excitation in both systems due to the presence of the core hole (see Fig. 8(a) and (d)). This observation will be important for the later discussion of the edge shift.

The RCD and RSD analyses of the initial ground and final core-excited states have provided valuable insight into the L-edge XAS process for each system. However, to understand the L-edge XAS shift requires a closer comparison of the effects of 2p–3d excitation in different oxidation states.

### Contraction of the 3d shell, increase of electron affinity in the final core-excited states and L-edge XAS shift

**Fig. 9:**
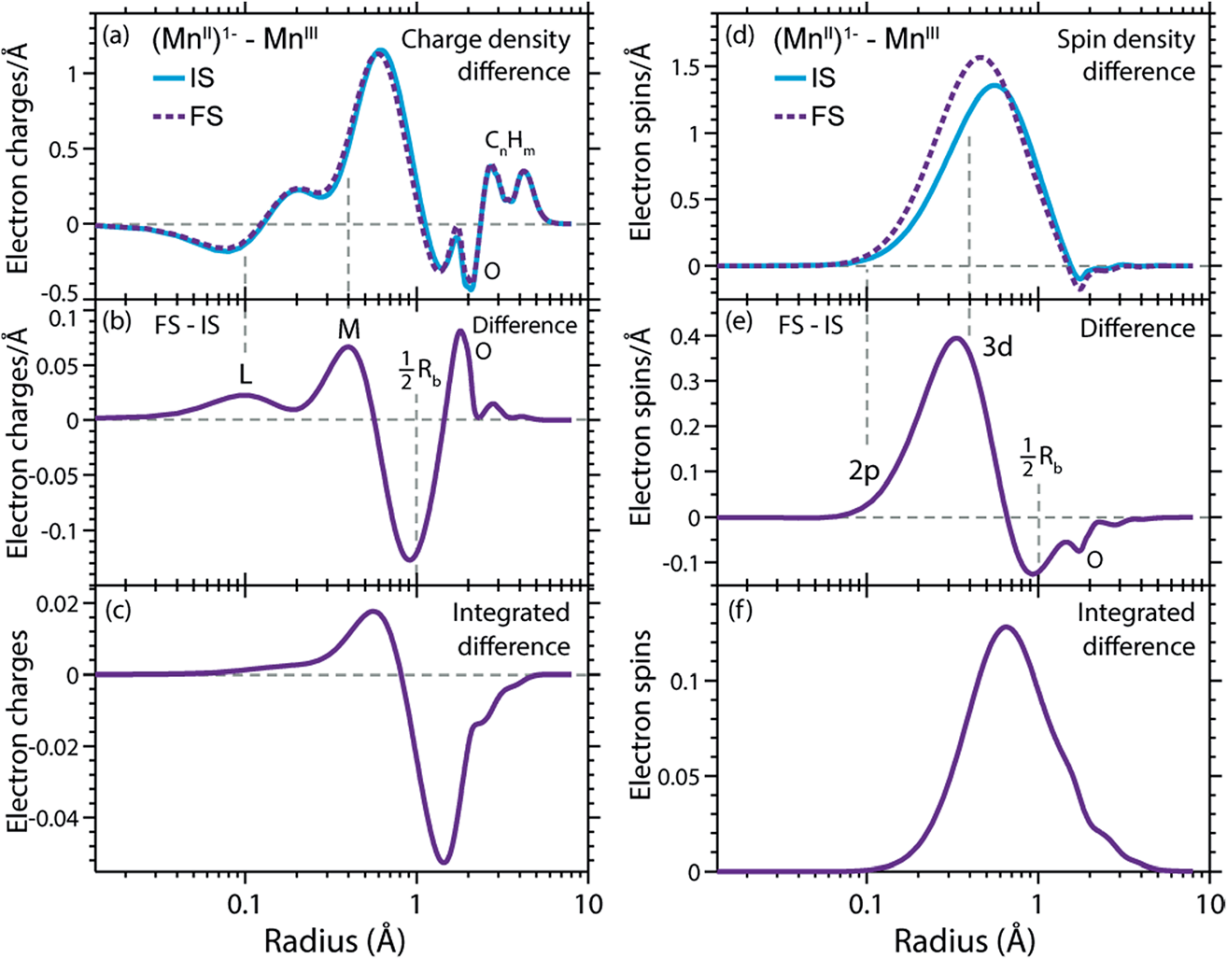
(a) Radial charge density (RCD) differences (RCD of (Mn^II^(acac)_3_)^1–^ minus RCD of Mn^III^(acac)_3_) in the initial ground states (IS) and in the final core-excited states (FS). (b) Difference of the RCD differences between initial ground and final core-excited states (RCD difference from (a) between (Mn^II^(acac)_3_)^1–^ and Mn^III^(acac)_3_ in the final core-excited states minus RCD difference from (a) between (Mn^II^(acac)_3_)^1–^ and Mn^III^(acac)_3_ in the initial ground state). (c) Integrated difference of RCD differences in (b)). (d–f) Corresponding plots for the radial spin density (RSD), with FS densities averaged over selected states in the L_3_-edge with Δ*S* = 0 (quintet for Mn^III^ and sextet for Mn^II^). The dashed vertical lines indicate the location of L and M shell maxima and 2p and 3d shell maxima in (a–c) and (d–f), respectively, and approximately half the Mn–O bond length *R*_b_.

In Fig. 9(a) and (d) we show difference curves of the RCDs and RSDs of (Mn^II^(acac)_3_)^1–^ minus Mn^III^(acac)_3_ for the initial ground states and the final core-excited states, respectively. The ground-state curves are identical to those in Fig. 5 (now on a logarithmic scale for the radius) and show only minor differences between Mn^II^ and Mn^III^. The most obvious difference between the two systems is seen for the RSDs in Fig. 9(d), where the maximum in the RSD difference lies 0.1 Å closer to the Mn nucleus in the final core-excited states compared to the initial ground states. With the added spin reflecting the extra 3d electron, core excitation thus puts this electron closer to the nucleus. In order to balance the shift of the extra electron in the 3d shell towards the Mn nucleus in the final states, other electrons in the doubly occupied orbitals are polarized such that charge density is pushed away from Mn towards the oxygen ligands (Fig. 9(b and c)).

This allows us to establish a qualitative explanation of the L-edge XAS shift: the important observation is that the peak in the spin density of the additional electron in the 3d shell (Fig. 9(d)) is shifted by 0.1 Å to smaller radii upon 2p–3d excitation. With the extra 3d electron added closer to the nuclei, we naturally interpret this as corresponding to a lower energy for that state, or alternatively, as a higher electron affinity in the final core-excited states as compared to the initial ground state. Although the polarization of the other electrons may partially counteract this effect, the net result is still an increase in electron affinity for the core-excited state.

**Fig. 10:**
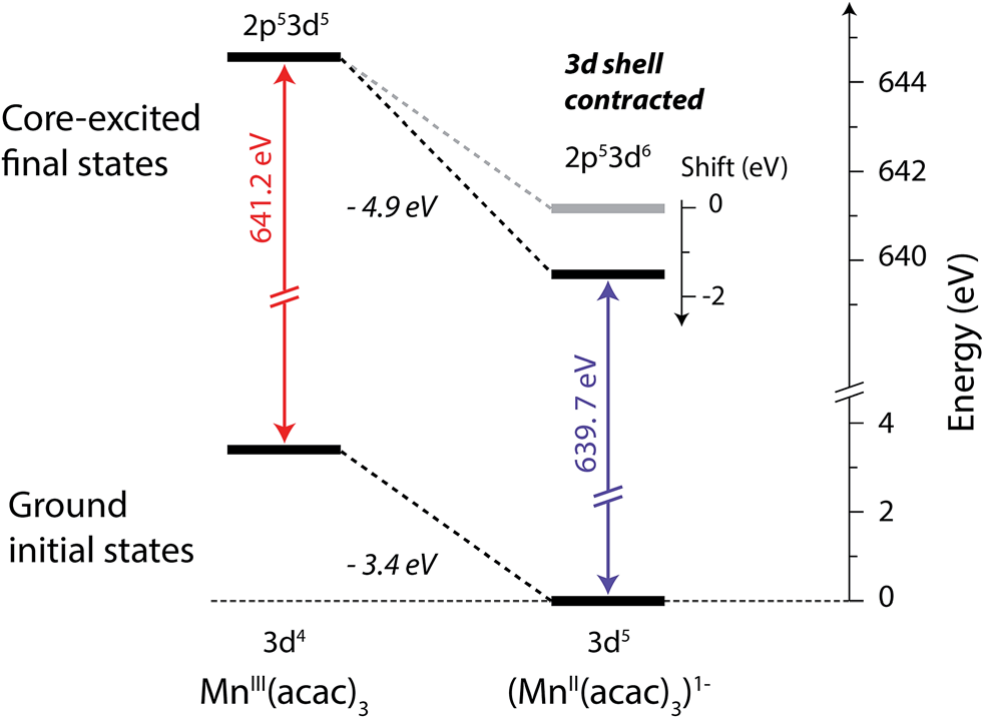
Total-energy level diagram of Mn^III^(acac)_3_ and (Mn^II^(acac)_3_)^1–^ for calculated initial ground and final core-excited states. The difference in the ground state energies of (Mn^II^(acac)_3_)^1–^ and Mn^III^(acac)_3_ was directly extracted from the RAS calculations. The final core-excited state energies are the RAS calculated L_3_-edge absorption maxima for the multiplicity components with Δ*S* = 0 (quintet for Mn^III^ and sextet for Mn^II^, see Table 1).

The relation between the electron affinity and the L-edge XAS shift is outlined in a total energy scheme (Fig. 10). The lower ground-state energy of (Mn^II^(acac)_3_)^1–^ as compared to Mn^III^(acac)_3_ represents an “attractive” affinity of 3.4 eV for Mn^III^(acac)_3_ towards binding an additional electron (Fig. 10). The same analysis for the core-excited states in the L_3_ maxima gives, as predicted above, a higher affinity (4.9 eV). These values underestimate the real electron affinity, as the geometry of the reduced state is not allowed to relax. However, this error should cancel when comparing differences of state energies. The energy required to reach the final core-excited states from the initial ground state, *i.e.*, the XAS L-edge energies (vertical arrows in Fig. 10) is thus lower for the reduced complex. In this analysis, the difference in electron affinity of +1.5 eV is thus identical to the L-edge shift.

### L-edge XAS shift between Mn^II^(acac)_2_ and Mn^III^(acac)_3_

The validity of transferring arguments and conclusions from the (Mn^II^(acac)_3_)^1–^-Mn^III^(acac)_3_ comparison to the Mn^II^(acac)_2_–Mn^III^(acac)_3_ comparison is shown in Fig. S4 in the ESI,† where we compare the charge (RCD) and spin density (RSD) differences between initial ground and final core-excited states for the three systems addressed here. The overall picture is the same for both coordination environments of the reduced complex, with differences in charge and spin density distributions on the level of 0.01–0.02 electron charges and 0.03 electron spins. This is consistent with the similarity in spectra and relative L-edge absorption energies, and supports that our explanation for the L-edge XAS shift applies equally for the shift between spectra of Mn^II^(acac)_2_ and Mn^III^(acac)_3_.

### Connections to earlier findings on L-edge XAS shape and shift

With our detailed description of the 2p–3d excitation process in both reduced and oxidized states and in initial ground and final core-excited states, we can now connect our claims to previous rationalizations of changes in spectral shape and energy shift with oxidation state. We do this by evaluating the most relevant 2p–3d and 3d–3d Coulomb interactions by inspecting the pairwise electron interactions for the electron or spin configurations extracted from the observed changes in charge and spin distributions. We note, however, that due to the extensive changes in radial distributions for both oxidation-state change and core excitation (Fig. 7–9) future and more extensive analyses will have to detail the changes in the numerous pairwise interactions to ultimately distinguish and more exactly quantify their respective contributions to the L-edge XAS shift. Here we just estimate the various contributions to the shift to indicate some of the most important consequences of our findings and how they relate to previous explanations of the shift.

Further, the spin analyses in Fig. 4 illustrate how the spectral shape is affected by the relative energy of spin configurations with different multiplicity or number of parallel spins. Considering the near atomic nature of the spin distribution in all systems revealed by the RSD plots, it is clear that multiplet and ligand-field theory can be used to explain the main features of the spectral shape. As an example, the larger spectral width of the L_3_-edge of Mn^III^(acac)_3_ compared to Mn^II^(acac)_2_ is due to the larger multiplet splitting of the final states in Mn^III^ 2p^5^3d^5^ quintet (Δ*S* = 0) compared to Mn^II^ 2p^5^3d^6^ sextet (Δ*S* = 0) (ref. 35). This is an important consistency test to see this fundamental intra-atomic property to be correctly reflected in the molecular RAS calculations.

Most importantly, we have identified the dominant spin configurations in the final core-excited states with Δ*S* = 0 (sextet in Mn^II^ and quintet in Mn^III^) to the ones with parallel 2p and 3d spins for both systems (see insets in Fig. 8(b) and (e)). Upon transitions from the initial to these final states the changes in the number of 2p–3d and 3d–3d pairwise electron interactions with parallel spin is the same for both Mn^II^ and Mn^III^ (compare the number of exchange integrals K in initial and final states for Mn^II^ and Mn^III^ in Table S1†). Contributions of exchange interactions to the shift between the L_3_-edge absorption maxima of Mn^II^ and Mn^III^ can, therefore, to first approximation, be neglected. This therefore excludes the notion of a varying number of unpaired 3d electrons dominating the L-edge XAS shift and reduces the atomic analysis to the direct Coulomb interactions.

Instead, counting the changes in the number of all electron pairs (direct Coulomb integrals) reveals that the contribution to the Mn^II^–Mn^III^ L-edge XAS shift is one extra 2p–3d interaction and one less 3d–3d interaction (compare the number of Coulomb integrals *J* in initial and final states for Mn^II^ and Mn^III^ in Table S1†). As the 2p–3d Coulomb interactions (Q) are larger than the 3d–3d Coulomb interactions (U) (see Table S2†) the L_3_-edge shift is to higher energies for Mn^III^ compared to Mn^II^. This particular contribution to the L-edge XAS shift amounts to the “Q–U explanation” of the shift with oxidation-state change by van der Laan and Kirkman.^26^ In addition to changes in the number of interactions, the size of each interaction is different in initial and final states, and also between oxidation states. This will also affect the relative energies. Still, as the (direct) Coulomb interactions are about one order of magnitude larger than the exchange interactions (see Table S2†), we find that within the restrictions made here (transitions Δ*S* = 0 final states and by counting the corresponding changes in pairwise electron interactions) the oxidation-state dependent L-edge XAS shift is dominated by differences in direct Coulomb interactions in the final core-excited states. The same analysis for the L_3_-edge shift between Mn^III^ and Mn^IV^, gives the same result (negligible contribution of exchange interactions and dominance of direct Coulomb interactions), consistent with experimentally observed shifts of 1.5–2 eV for both Mn^III^ to Mn^IV^ and Mn^II^ to Mn^III^.^22^,^25^

Alternatively, if the shift was due to increased screening of the 2p core hole by the extra electron in the reduced complex, the expected effect would be a displacement of the 2p spin density in the final state towards larger radius in the RSD plots in Fig. 9(d–f). Such a shift cannot be seen. As outlined in Materials and methods, our calculations do not properly account for relaxation of the 2p shell during the core-excitation process state. However, significant screening by the added electron would have increased the 2p radius already in the ground state. There are changes in the electron density at short distances upon reduction (Fig. 9(a)), but these changes seems to be too small to have a significant effect on the 2p radial distribution. Considering the radial distributions, the extra 3d electron has a low probability of being between the 2p hole and the nuclei, and the screening of the hole is therefore small and possibly counteracted by the expansion of the M shell. Accurate quantification of these effects would require a detailed analysis using a more flexible treatment of the 2p orbitals, but at this level there is no evidence that the 2p shell is significantly affected by the reduction process. This argues against core hole screening as the dominant contribution to the spectral shift. Using similar arguments, the proposed large effect of the 2p–3d excitation on the extra 3d electron in the reduced complex can also be rationalized. The 2p hole effectively acts as an additional proton, and the excited electron in the 3d shell only partially screens this interaction, leading to significant changes in the position of the extra 3d electron.

The concept of effective nuclear charge *Z*_eff_^83^ conceptually overlaps with our findings, and it would be interesting to find a way to explicitly calculate this property in the final core-excited states. However, considering the large differences in electron–electron interactions in ground and excited states, such a calculation does not appear to be straightforward.

In addition to changes in oxidation state, there are other factors that also lead to L-edge shifts. Fe^II^ systems that can exist in different spin states exhibit a distinct shift to lower energy for the high-spin compared to the low-spin state.^49^ For low-spin iron systems like the hexacyanides, it has been shown that changes in oxidation state lead to a change in the ligand-field strength that is directly reflected in the shift.^83^ In our analysis we have isolated the basic origin of the oxidation-state shift by deliberately choosing ionic complexes where the effect of the ligand environment is relatively small. Here high-spin d^4^ and d^5^ complexes are ideal because changes in the ligand field have relatively small effects on the average energy of the configuration. The ground states of two complexes with neighboring oxidation states as chosen here inevitably have different spin states. By focusing on the Δ*S* = 0 component in the L-edge absorption process, however, the changes in the number of exchange interactions upon 2p–3d excitation is the same for the different oxidation states thereby uniquely connecting the L-edge XAS shift to changes in the oxidation state. Finally, the L-edge energies can also be affected by the coordination number, as shown for iron tetra- and hexachlorides.^19^ We estimated this and accounted for this effect here by introducing an artificial six-coordinate Mn^II^ complex, an approach made possible by the *ab initio* RAS simulations. This enables us to dissect the problem and focus here on the basic origin of the L-edge shift with changes in the oxidation state.

An important future test of our approach would be to apply it to high-spin Fe^II^ (d^6^) and Fe^III^ (d^5^) compounds^19^,^23^ as well as complexes with strong ligand fields where there are significant changes in metal–ligand interaction in different oxidation states.^83^,^84^ It may also be useful to analyze charge- and spin-density changes along specific bonds in the complex to reconcile the observations made here with established concepts on how coordination number and structure affect the L-edge XAS shift. To further asses the importance of core–hole screening it will in addition be interesting to compare charge and spin densities in the final states of L-edge XAS and XPS (X-ray photoelectron spectroscopy) as in XPS “the initial ground-state valence electrons experience the full potential of an unscreened core hole”.^82^

For 3d transition metal XAS or XANES we here uniquely relate charge and spin density changes in the initial ground and final core-excited states. This is of paramount importance not only to ascertain the information content of metal L-edge absorption spectroscopy. More importantly it explicitly tells us how charge and spin “are spread in space” at and around the metal. In future applications our approach may thus prove useful in predicting and probing how metal charge and spin densities change in photocatalytic reactions, in photochemical reactions and in biological processes involving metalloproteins. This will be essential to understand and ultimately control chemical reactivity in redox reactions of transition-metal systems.

Considering that the approach presented here gives an integrated picture of changes in spin and charge density, it would also be interesting to combine it with EPR spectroscopy that gives complementary information about the relation between spin and formal oxidation state.

## Summary and conclusions

We present a combined experimental and theoretical investigation of Mn L-edge absorption spectra of Mn^II^(acac)_2_ and Mn^III^(acac)_3_ in solution. The spectra were measured in a protocol that avoids X-ray induced sample damage in partial fluorescence yield mode and show the well-known and distinct increase in L-edge absorption energy (L-edge XAS shift) from Mn^II^(acac)_2_ to Mn^III^(acac)_3_ indicating the increasing oxidation state of the system. The experimental spectra are compared to spectra and radial charge and spin density distributions calculated with the restricted active space (RAS) molecular orbital method. With these *ab initio* calculations we uncouple the discussion of the L-edge XAS shift from the possible influence of the geometry of the complexes and focus exclusively on the role of charge and spin density changes at the metal sites for the spectral shift. Our results validate the picture from quantum chemical calculations that formal oxidation (or reduction) does not lead to distinct changes in metal charge. In accordance with this picture we demonstrate how L-edge XAS probes the electronic structure locally at the metal site in terms of changes in charge and spin density changes.

For the chosen high-spin systems with weak ligand fields, the L-edge absorption energies and the shape of the spectra are largely determined by atomic multiplet effects in the final core-excited states. The incident energy shift of 2.0 eV, quantified by the L_3_-edge maximum, is reproduced by RAS within 0.3 eV. This good agreement makes it for the first time possible to study changes in electronic structure for different redox states and core excitation on an equal level of approximation using an accurate molecular-orbital method. We do this through a detailed analysis of radial distributions of charge and spin densities in the initial ground and final core-excited states of the systems.

In agreement with earlier studies of ground and valence excited states of metal complexes, we find that upon reduction of Mn^III^ to Mn^II^ the number of electrons in the Mn 3d shell and thus the local spin density increases by unity. In contrast, the local charge at the Mn atom increases only by a fraction of an electron and the remaining charge is distributed over the ligands.

In the Mn systems studied here, we find the charge and spin densities at distances beyond the Mn 3d shell, namely in the bonds with the ligands, to react only to a negligible degree to 2p-3d excitation. This shows, in agreement with common notions, that after core-excitation the charge missing in the 2p shell is almost perfectly self-screened towards the ligands by the additional core-excited 3d electron. There is no evidence that the 2p shell is significantly affected by the reduction process. This argues against core hole screening as the dominant contribution to the spectral shift. We thus visualize, validate and quantify fundamental notions of L-edge XAS of 3d transition-metal systems and propose an improved description of how charge and spin densities are reflected in the spectra beyond the notation of formal oxidation states.

Effects on the 3d shell are found to be more significant. The differences in spin density distributions exhibit a shift by 0.1 Å towards the Mn nucleus in the final core-excited states compared to the initial ground states, interpreted as a compression of the 3d orbitals. We associate this contraction of the 3d shell in the core-excited states with an increased electron affinity of the metal complex. This decreases the energy required to reach the core-excited states of the reduced species and gives a chemically intuitive picture of the L-edge XAS shift. The shift is due to changes in the direct (classical) Coulomb interactions in the final states when changing the nominal oxidation state.

Given the still qualitative nature of our explanation of the L-edge shift, the next step is to exactly quantify the predominant interactions and their energy contributions relevant to the L-edge absorption shift, based on RAS calculations, by, *e.g.*, dissecting the shift into individual energy contributions of exchange energy and of direct Coulomb terms for all relevant electron–electron and electron–nuclei interactions. This remains an appealing target for future studies. We furthermore expect that more specific analyses of charge and spin contributions, *e.g.* along specified bond axes in the molecule, provide further insight into how charge and spin density changes determine L-edge absorption energies. Important future extensions may also include the study of Fe high-spin systems to assess in more detail the relative importance of symmetry or structure and oxidation state, as well as investigations of complexes with stronger ligand fields with significant changes in metal–ligand interaction in different oxidation states. It will also be important to extend our unified picture of how charge and spin densities change upon oxidation changes to other important techniques such as X-ray photoelectron spectroscopy and EPR.

We expect that our unified approach of explicitly relating the L-edge absorption spectra of 3d transition-metals to their local charge and spin densities will be applicable to a large number of inorganic metal complexes, metalloproteins and transition-metal catalysts. The conceptual innovation presented here uniquely relates changes in the valence electronic structure as probed by L-edge absorption spectroscopy to changes of charge and spin densities around the metal site and thus reveals how changes in the formal metal oxidation state determine L-edge absorption energies. Knowing how charge and spin “are spread in space” at and around the metal is essential for a mechanistic understanding of photocatalytic and photochemical reactions and of biological function of metalloproteins. The improved description of L-edge XAS presented here could build a new basis for understanding and ultimately controlling redox-catalytic reactions of molecular complexes and metalloproteins.

## Author contributions

Conceived the experiment: M. K., V. K. Y., J. Y., U. B., Ph. W. Planned the experiment: M. K., J. K., Ph. W., R. M. Prepared the samples: M. K. Carried out the experiment: M. K., H. L., R. M. Analyzed the experimental data: M. K., Ph. W. Designed the calculations: M. G., M. L. Carried out the calculations: M. G., E. K., M. L. Analyzed the calculations: M. G., M. K., Ph. W., M. L. All authors discussed the results. Wrote the paper: M. K., M. L. and Ph. W. with contributions from all authors.

## Conflicts of interest

There are no conflicts of interest to declare.

## Supplementary Material

Supplementary information
