# Supplementary material for: Probing the oxidation state of transition metal complexes: a case study on how charge and spin densities determine Mn L-edge X-ray absorption energies
Source: Chem Sci. 2018 Jul 17;9(33):6813–29. doi: 10.1039/c8sc00550h (PMC6115617; doi:10.1039/c8sc00550h)
Supplement: Supplementary file 1 [file SC-009-C8SC00550H-s001.pdf]

Electronic Supplementary Material (ESI) for Chemical Science.
This journal is © The Royal Society of Chemistry 2018

Supporting Information

Probing the Oxidation State of Transition Metal Complexes: A Case Study on How Charge and Spin Densities Determine Mn L-Edge X-ray Absorption Energies

Markus Kubin[1#], Meiyuan Guo[2#⊥], Thomas Kroll[3], Heike Löchel[4], Erik Källman[2], Michael L. Baker[5], Rolf Mitzner[1], Sheraz Gul[6], Jan Kern[6, 7], Alexander Föhlisch[1, 8], Alexei Erko[4], Uwe Bergmann[9], Vittal Yachandra[6], Junko Yano[6], Marcus Lundberg[2]*, Philippe Wernet[1]*

[1]Institute for Methods and Instrumentation for Synchrotron Radiation Research, Helmholtz-Zentrum Berlin für Materialien und Energie GmbH, Albert-Einstein-Strasse 15, 12489 Berlin, Germany

[2]Department of Chemistry-Ångström Laboratory, Uppsala University, Sweden

[3]SSRL, SLAC National Accelerator Laboratory, California 94025, Menlo Park, USA

[4]Institute for Nanometre Optics and Technology, Helmholtz-Zentrum Berlin für Materialien und Energie GmbH, Albert-Einstein-Strasse 15, 12489 Berlin, Germany

[5]The school of Chemistry, The University of Manchester at Harwell, Didcot, OX11 OFA, UK

[6]Molecular Biophysics and Integrated Bioimaging Division, Lawrence Berkeley National Laboratory, Berkeley, California 94720, USA

[7]LCLS, SLAC National Accelerator Laboratory, Menlo Park, California 94025, USA

[8]Institut für Physik und Astronomie, Universität Potsdam, Karl-Liebknecht-Strasse 24/25, 14476 Potsdam, Germany

[9]Stanford PULSE Institute, SLAC National Accelerator Laboratory, Menlo Park, California 94025, USA

[#]These authors contributed equally to this work.

[⊥]Current address: Key Laboratory of Luminescence and Real-Time Analytical Chemistry (Southwest University), Ministry of Education, College of Chemistry and Chemical Engineering, Southwest University, Chongqing 400715, China.

*Authors to whom correspondence should be addressed: marcus.lundberg@kemi.uu.se, wernet@helmholtz-berlin.de

X-ray Dose Accumulated in a Probed Sample Volume

X-ray dose is the ratio of absorbed energy and absorbing mass. For flowing solution samples the choice of the absorbing volume and thus mass is often ambiguous. In order to provide an upper limit for the dose absorbed under our experimental conditions we herein calculate the "skin dose" (see refs. [22,40] in the main paper). This is the dose absorbed by a sample volume spanned by the surface area that exposed to the x-ray beam and the attenuation length Λ of the sample (here Λ=1.44 µm for ethanol and Λ=1.14 µm for acetylacetone at 640 eV photon energy). We experimentally determined the horizontal liquid jet diameter to d_{jet}=20±4 µm and 22±4 µm for the $Mn^{II}(acac)_2$ and $Mn^{III}(acac)_3$ solution samples, respectively, the x-ray focus size to $H\times V$=100×90 µm² (horizontal×vertical, FWHM), and the beamline flux to Φ=(5.4±0.1)x10^{12} photons/s at hν=640 eV photon energy. Only a portion $\eta \approx d_{jet}/H$ of the photons hit the sample due to the smaller horizontal size of the liquid jet as compared to the horizontal focus size. For the $Mn^{II}(acac)_2$ and $Mn^{III}(acac)_3$ solution samples we measured sample flow rates F of 8.5±1.5 µl/min and 12±0.1 µl/min, respectively, corresponding to jet velocities $v_{jet}=4F/(\pi\cdot d_{jet}^2)$=0.45±0.20 m/s and 0.53±0.20 m/s and illumination times $t=V/v_{jet}$=200±90 µs and 170±70 µs, respectively. The resulting skin dose D_s is calculated via

$$D_s = \frac{\Phi \cdot \eta \cdot \mathrm{h\nu} \cdot \mathrm{t} \cdot (1 - 1/\mathrm{e}) \cdot 0.761}{\rho \cdot \Lambda \cdot d_{jet} \cdot \mathrm{V}} = \frac{\Phi \cdot \mathrm{h\nu} \cdot \mathrm{d}_{\mathrm{jet}}^2 \cdot \pi \cdot (1 - 1/\mathrm{e}) \cdot 0.761}{4 \cdot F \cdot H \cdot \rho \cdot \Lambda}$$

where we used the above defined identities and where the factors (1-1/e) and 0.761 account for the portions of photons absorbed within Λ and contained within the vertical focus size V (FWHM), respectively. With ρ(ethanol)=789 kg/m³ and ρ(acetylacetone)=980 kg/m³ the skin doses estimated for solution samples of $Mn^{II}(acac)_2$ in ethanol and $Mn^{III}(acac)_3$ in acetylacetone are 5200±2300 Gy and 4600±1700 Gy, respectively (1 Gy= 1 J/kg).

Structures and Orbitals of $Mn^{II}(acac)_2$, $Mn^{III}(acac)_3$, and $(Mn^{II}(acac)_3)^{1-}$

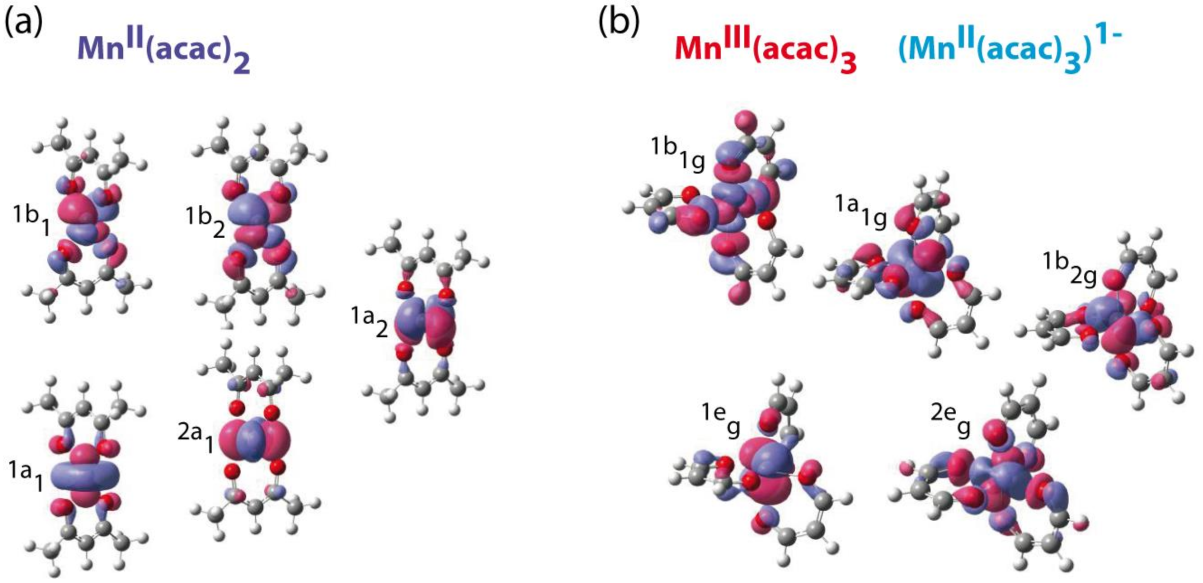

Figure S1. Calculated plots of selected valence orbitals of (a) $Mn^{II}(acac)_2$ in T_d and C_{2v} symmetry and of (b) $Mn^{III}(acac)_3$ and $(Mn^{II}(acac)_3)^{1-}$ in O_h and D_{4h} symmetry. Details of the calculations can be found in the Methods and Materials section of the main paper. Different phases of the orbitals (derived from RAS calculations, representing the active space for calculated RAS spectra) are shown in blue and red and orbitals are displayed with an isovalue of 0.02 au.

For the approximately tetrahedral (T_d) $Mn^{II}(acac)_2$ complex we find for the four Mn-O bonds, Mn to O distances of 2.06 Å (Figure 1 (a) in the main paper). The point group is D_{2d} but as MOLCAS only handles Abelian point groups, the subgroup C_{2v} is used for all calculations and for labeling the orbitals. The six-coordinated $Mn^{III}(acac)_3$ complex has C_2 symmetry and the octahedral ligand environment is affected by a strong Jahn-Teller distortion (refs. Forman (1959) and Stults (1979) in this document) leading to approximate D_{4h} symmetry (Figure 1 (b) in the main paper).

Compared to the published x-ray structure of $Mn^{III}(acac)_3$ (ref. Stults (1979) in this document), the deviation between Mn-O distances is only 0.01 Å, except along the Jahn-Teller axis where the distance is overestimated by 0.05 Å. For $Mn^{II}(acac)_2$ we are not aware of any crystallographic data since the compound crystallizes as $Mn^{II}(acac)_2$ trimers (ref. Shibata (1984) in this document) resulting in a six-coordination of each Mn with Mn-O bond lengths between 2.1 and 2.2 Å. The Mn-O bond length of 2.06 Å from DFT optimization for $Mn^{II}(acac)_2$ (Figure 1 (a) in the main paper) is consistent with this. The difference in Mn-O bond lengths of $Mn^{II}(acac)_2$ and $Mn^{III}(acac)_3$ as well as the Jahn-Teller distortion of $Mn^{III}(acac)_3$ are also reflected in the results of infrared (IR) spectroscopy, published in refs. Forman (1959) and Shapkin (2006).

Decomposition of the RAS Spectra into Orbital Components

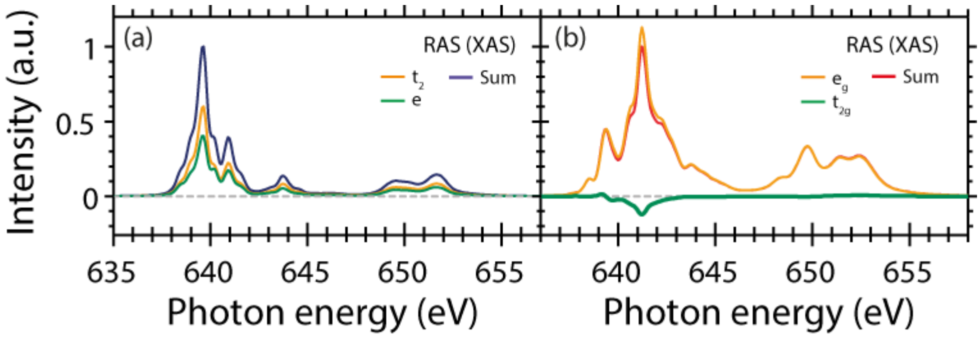

Figure S2. Calculated RAS absorption spectra (XAS not PFY-XAS) decomposed according to the relative orbital contributions in the final core-excited states for (a) $Mn^{II}(acac)_2$ (T_d labels) and (b) $Mn^{III}(acac)_3$ (O_h labels).

In $Mn^{II}(acac)_2$ from the sextet $e^2t_2^3$ (T_d) ground state, excitations to both e and t_2 orbitals contribute with similar relative intensity over almost the entire range of the spectrum (Figure S2 (a)). The one-electron picture of excitations to distinct orbitals is consequently not appropriate for interpreting individual features. The orbital analysis of $Mn^{III}(acac)_3$ ($t_{2g}^3e_g^1$ in O_h) looks different (exclusive excitation to e_g and partial depopulation of t_{2g}, Figure S2 (b)), but the conclusion is very similar: Strong mixing of different electron configurations in the final states gives close to unity occupation numbers for all orbitals over the entire spectral range, which corresponds to a change in occupation number of zero for all orbitals except for the initially unoccupied e_g (b_{1g} or $d_{x2-y2,}$ see Figure 1 (b) of the main paper) orbital in $Mn^{III}(acac)_3$ where it is unity.

Spin Multiplicity Analysis of $(Mn^{II}(acac)_3)^{1-}$

We further plot in Figure S3 the calculated RAS spectrum of $(Mn^{II}(acac)_3)^{1-}$ decomposed according to the spin multiplicity components sextet, quartet, and doublet. As could be expected, the spin multiplicity analysis of the $(Mn^{II}(acac)_3)^{1-}$ RAS spectrum also gives very similar results as for $Mn^{II}(acac)_2$ (Figure 4 (c) in the main paper).

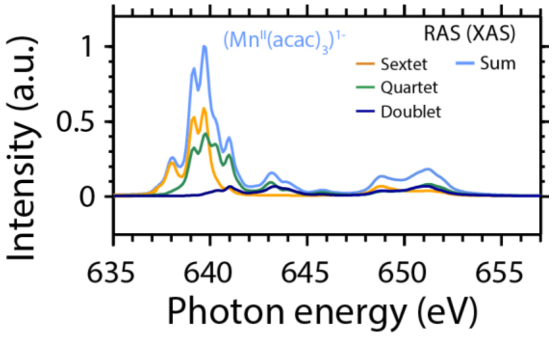

Figure S3. Contribution of (spin) multiplicities 2S+1 in the core-excited final XAS states for $(Mn^{II}(acac)_3)^{-1}$ as obtained from the RAS calculations. The absolute photon energies of the spectrum are calibrated as in the main paper.

Initial Ground-State and Core-Excited State Configurations for Mn^{II} and Mn^{III} with ΔS=0 within the LS Coupling Scheme and Number of Coulomb and Exchange Integrals

Table S1. Electron spin configurations and number of Coulomb (J) and exchange (K) integrals of the Mn^{II} $3d^5$ and Mn^{III} $3d^4$ initial ground state configurations and the Mn^{II} $2p^53d^6$ and Mn^{III} $2p^53d^5$ final core-excited state configurations (black: paired spins, red: unpaired spins).

	Mn(II)		Mn(III)	
Initial State	Sextet<br>$2p^6$ (^1S) $3d^5$ (^6S) ^6S<br>↑↓ ↑↓ ↑↓ ↑ ↑ ↑ ↑ ↑	30 x J_{pd}<br>10 x J_{dd}<br>15 x K_{pd}<br>10 x K_{dd}	Quintet<br>$2p^6$ (^1S) $3d^4$ (^5D) ^5D<br>↑↓ ↑↓ ↑↓ ↑ ↑ ↑ ↑	24 x J_{pd}<br>6 x J_{dd}<br>12 x K_{pd}<br>6 x K_{dd}
Final State	$2p^5$ $3d^6$		$2p^5$ $3d^5$	
ΔS= 0	Sextet<br>$2p^5$ (^2P) $3d^6$ (^5D) ^6L<br>↑↓ ↑↓ ↑ ↑ ↑ ↑ ↑ ↑ ↑↓	30 x J_{pd}<br>15 x J_{dd}<br>17 x K_{pd}<br>10 x K_{dd}	Quintet<br>$2p^5$ (^2P) $3d^5$ (^4L) ^5L<br>↑↓ ↑↓ ↑ ↑ ↑ ↑ ↑ ↓	25 x J_{pd}<br>10 x J_{dd}<br>14 x K_{pd}<br>6 x K_{dd}
			$2p^5$ (^2P) $3d^5$ (^6S) ^5P<br>↑↓ ↑↓ ↓ ↑ ↑ ↑ ↑ ↑	25 x J_{pd}<br>10 x J_{dd}<br>10 x K_{pd}<br>10 x K_{dd}

Slater Integrals

To quantify the energies of different configurations we use Coulomb (J) and exchange (K) integrals, where the total interaction between electrons of opposite spin is J, and the interaction between electrons of parallel spin is (J-K). As the effect of the ligand environment is small for the complexes studied here, it is reasonable to start with an analysis based on atomic theory, in which case the integrals J and K can be derived from the Slater integrals for direct and exchange Coulomb interactions, F^k and G^k,(ref. Slater (1929)) in the terminology of R. D. Cowan (ref.[78] of the main text). In Table S2 we list the average J and K integrals for ionic Mn^{II} and Mn^{III} initial ground and Mn^{II} $2p^53d^6$ and Mn^{III} $2p^53d^5$ final core-excited states for respective p (2p) and d (3d) electron interactions (the corresponding Slater integrals were also calculated and are identical to the values reported in ref.[26] of the main paper).

Table S2. Calculated energies of electron-electron interactions for Coulomb (J) and exchange (K) integrals for p (2p) and d (3d) electrons relevant to L-edge XAS of Mn^{II} and Mn^{III} for initial ground states (IS) and final core-excited states (FS). Averaged values of J and K integrals are given where averages were performed over all pairs of possible interactions in (d,d) and (p,d) orbitals.

Integral	Mn^{II} IS	Mn^{III} IS	Mn^{II} FS	Mn^{III} FS
**J(d,d)**	22.56	24.43	24.32	26.09
**J(p,d)**	31.34	33.31	33.63	35.47
**K(d,d)**	0.85	0.95	0.92	1.01
**K(p,d)**	0.70	0.81	0.84	0.94

The J and K integral values in Table S2 represent averages over interactions of electrons in all different tuples of the different orbitals spreading over ranges up to 9.6% (J(d, d)), 4.5% (J(p, d)), 65% (K(d, d)) and 162% (K(p, d)), relative to these average values. For example, in the excited states of Mn^{II} (Mn^{III}) the J integrals spread from 23.3 to 25.6 eV (24.9 to 27.4 eV) for J(d, d) and from 32.9 to 34.4 eV (34.7 to 36.3 eV) for J(p, d), while the K integrals spread from 0.55 to 1.15 eV (0.61 to 1.26 eV) for K(d, d) and from 0.16 to 1.52 eV (0.18 to 1.71 eV) for K(p, d).

The number of direct and exchange Coulomb interactions (number of J and K integrals) in the final core-excited states are given in Table S1. Together with the corresponding numbers in the initial ground states of the two systems (also given in Table S1) we can now calculate how the number of J and K integrals changes for going from initial to final states and for going from Mn^{II} to Mn^{III}. For Mn^{II} the 2p-3d excitation process changes the number of exchange Coulomb interactions K(p, d) and K(d, d) by 2 and 0, respectively (see Table S1). For Mn^{III} and upon 2p-3d excitation the number of K(p, d) and K(d, d) integrals as well changes by 2 and 0, respectively ($\Delta S=0$ final states with parallel 2p and 3d spins). The net difference of changes in the number of exchange interactions between Mn^{II} and Mn^{III} upon 2p-3d excitation is thus zero.

In Mn^{II} and upon 2p-3d excitation the number of direct Coulomb interactions J(p, d) and J(d, d) changes by 0 and 5, respectively, in (Table S1). For Mn^{III} the number of J(p, d) and J(d, d) integrals changes by 1 and 4, respectively. The contribution to the shift when going from Mn^{II} to Mn^{III} is then +1 J(p, d) and -1 J(d, d). As J(p, d)>J(d, d) (see Table S2) this qualitatively explains the L-edge XAS shift to higher energies for Mn^{III} compared to Mn^{II} (for the $\Delta S=0$ final states with parallel 2p and 3d spins).

Some of the observations from the RSD analysis can be directly related to the size of the Coulomb integrals. The smaller pairwise interactions in the initial ground state of Mn^{II} as compared to Mn^{III} (see Table S2) is directly related to the expansion of the 3d shell upon the addition of an extra electron (Figure 5 (d) in the main paper). In the same way, the contraction of the 3d shell upon core excitation (Figure 9 (d) in the main paper) is consistent with the

larger interactions for the final core-excited states relative to the initial ground states (Table S2, and ref.[26] in the main paper).

Comparison of Charge and Spin Density Differences for $Mn^{II}(acac)_2$ and $(Mn^{II}(acac)_3)^{1-}$

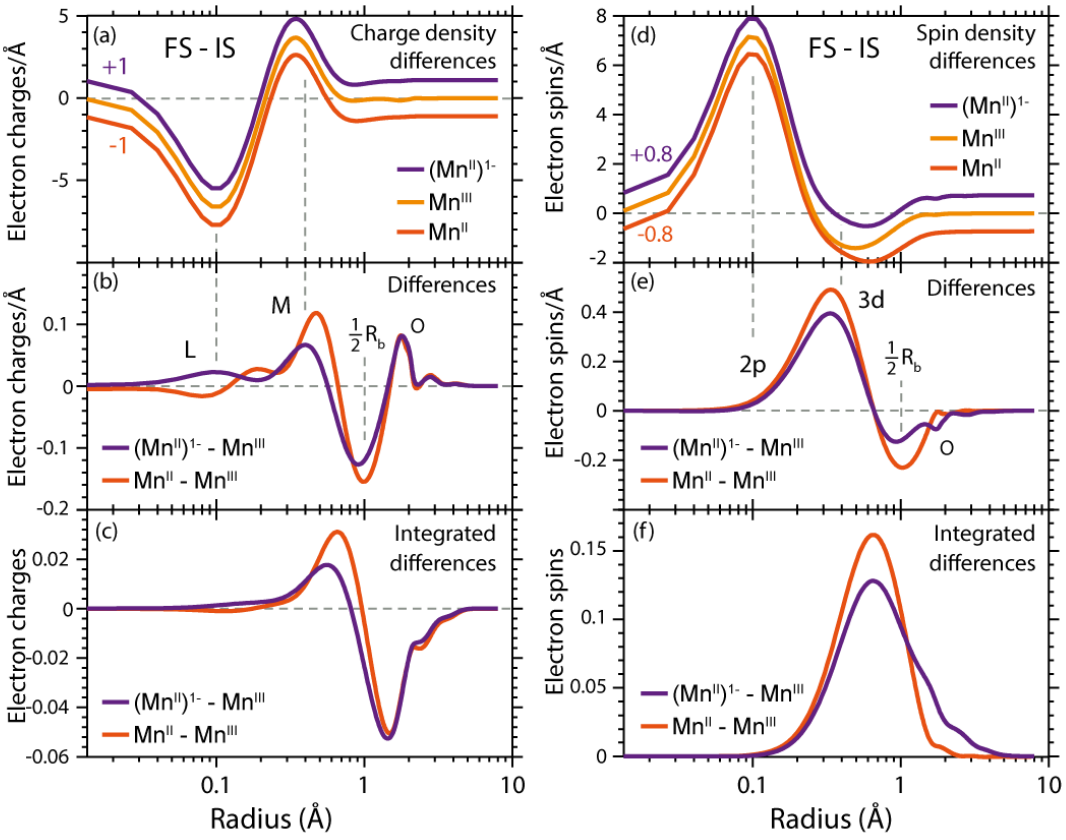

Figure S4. (a) Radial charge density (RCD) differences (RCD of core-excited final state minus RCD of ground initial state) of $(Mn^{II}(acac)_3)^{1-}$, $Mn^{II}(acac)_2$, and $Mn^{III}(acac)_3$ (in units of one electron charge per Å, averaged over all core-excited states). (b) Differences of RCD differences from (a) between $(Mn^{II}(acac)_3)^{-1}$ and $Mn^{III}(acac)_3$ and between $Mn^{II}(acac)_2$ and $Mn^{III}(acac)_3$. (c) Integrated RCD differences (integrals of the RCD differences from (b), in units of one electron charge). (d) Radial spin density (RSD) differences (RSD of core-excited state minus RSD of ground state) of $(Mn^{II}(acac)_3)^{1-}$, $Mn^{II}(acac)_2$, and $Mn^{III}(acac)_3$ (in units of one electron charge per Å, averaged over five selected core-excited states in the L_3-edge with $\Delta S=0$, namely sextet for $(Mn^{II}(acac)_3)^{-1}$ and $Mn^{II}(acac)_2$ and quintet for $Mn^{III}(acac)_3$). (e) Differences of RSD differences from (d) between $(Mn^{II}(acac)_3)^{-1}$ and $Mn^{III}(acac)_3$ and between $Mn^{II}(acac)_2$ and $Mn^{III}(acac)_3$. (f) Integrated RSD differences (integral of the RSD differences from (e), in units of one electron charge). All properties were extracted from our RAS calculations (see Methods and Materials section in the main paper) and are plotted versus the radius of a sphere around Mn. The dashed vertical lines indicate the location of L and M shell maxima in the charge distributions, of the 2p and 3d shell maxima in the spin density distributions and approximately half the Mn-O bond length R_b (some methyl groups were replaced by H atoms, see Figure 1 of the main text, with negligible effect on the calculated charge density distributions).

References of the Supporting Information

S1 A. Forman and L. E. Orgel, Molecular Physics, **1959**, 2, 362-366.

S2 B. R. Stults, R. O. Day, R. S. Marianelli and V. W. Day, Inorganic Chemistry, **1979**, 18, 1847-1852.

S3 S. Shibata, S. Onuma and H. Inoue, Chemistry Letters, **1984**, 13, 485-486.

S4 N. P. Shapkin, V. I. Razov, I. V. Tonkikh, I. V. Svistunova, A. S. Skobun and K. A. Goncharova, Russian Journal of Coordination Chemistry, **2006**, 32, 359-363.

S5 J. C. Slater, Physical Review, **1929**, 34, 1293-1322.
